# Supplementary material for: Structural basis of Gip1 for cytosolic sequestration of G protein in wide-range chemotaxis
Source: Nat Commun. 2018 Nov 6;9:4635. doi: 10.1038/s41467-018-07035-x (PMC6219514; doi:10.1038/s41467-018-07035-x)
Supplement: Supplementary file 1 — Supplementary Information [file 41467_2018_7035_MOESM1_ESM.pdf]

Supplementary information

Structural basis of the cytosolic sequestration of G protein by Gip1 in wide-range chemotaxis

Miyagawa et al.

This PDF file includes the following:

Supplementary Figure legends

Supplementary Figures 1 to 11

Supplementary Tables 1 to 3

Supplementary References (1-22)

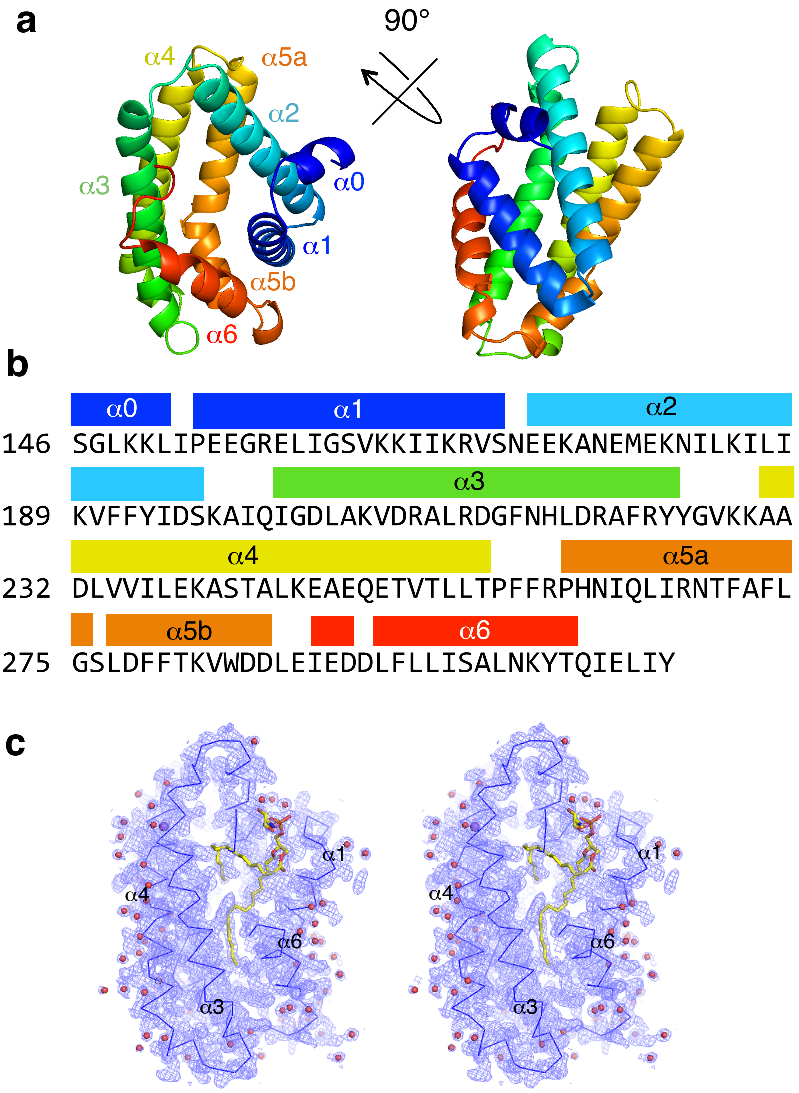

Supplementary Figure 1. Structural features of Gip1. (a) Cartoon representation of Gip1(146-310) viewed from the top (left) and side (right). (b) Secondary structure assignment of the sequence of Gip1(146-310). (c) A Stereo view of Gip1(146-310; Form I) (PDB 5Z1N). Overall structure is shown by a ribbon and stick model with the 2mFo-DFc electron density map contoured at 1.0 σ. Water molecules and a sodium ion are shown as red and purple balls, respectively.

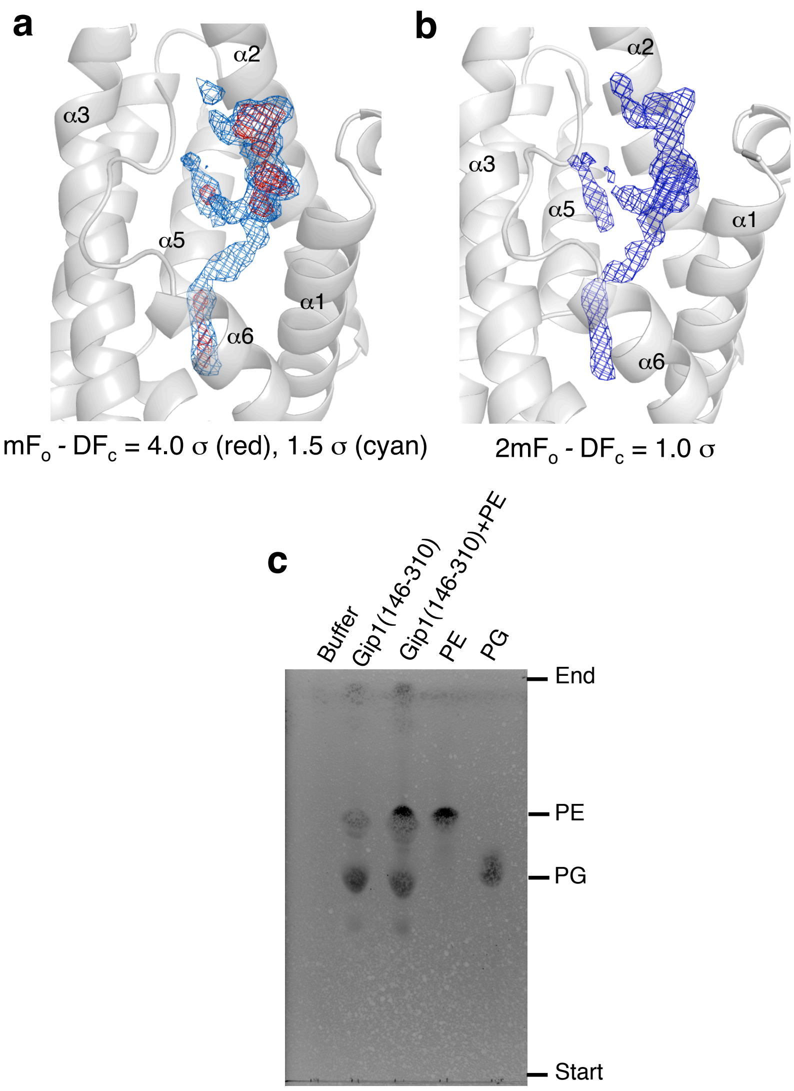

Supplementary Figure 2. Electron density in the hydrophobic cavity. (a) The structure of Gip1(146-310) with a possible glycerophospholipid. The mFo-DFc omit electron density map is contoured at 4.0 σ (red mesh) and 1.5 σ (cyan mesh). (b) The model structure of PE and PG with Gip1(146-310). The 2mFo-DFc electron density map of the phospholipid is contoured at 1.0 σ (blue mesh). (c) Determination of phospholipids in Gip1(146-310) by thin layer chromatography. Lipid extracts from buffer, Gip1(146-310) samples, PE and PG were separated and visualized on a silica

plate. Two major spots on the Gip1(146-310) lane were determined to be PE (upper) and PG (bottom) based on comparison with standards.

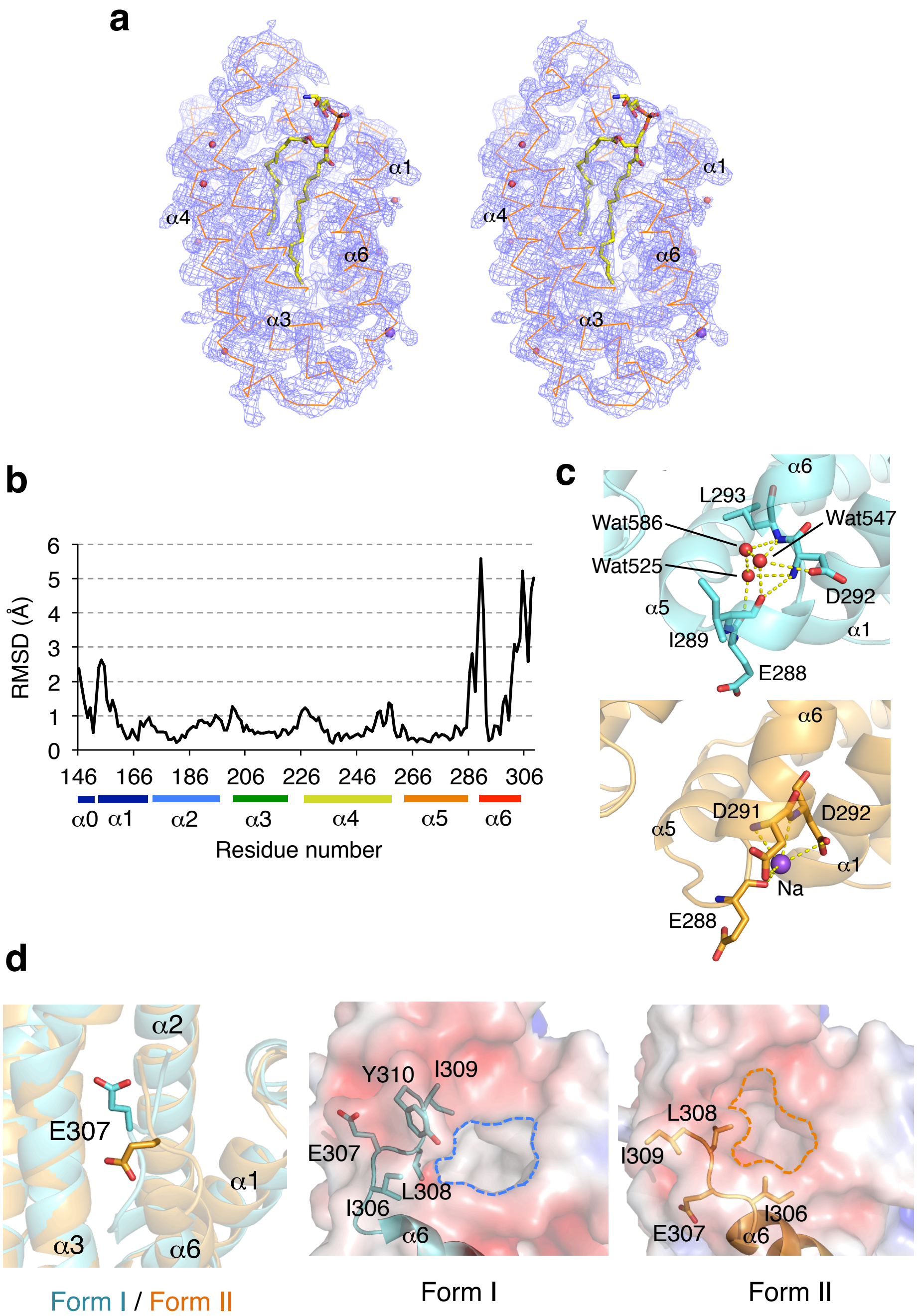
a
α1
α4
α6
α3
α1
α4
α6
α3
b
RMSD (Å)
6
5
4
3
2
1
0
146
166
186
206
226
246
266
286
306
α0
α1
α2
α3
α4
α5
α6
Residue number
c
α6
L293
Wat586
Wat547
Wat525
D292
α5
α1
I289
E288
α6
D291
D292
α5
α1
Na
E288
d
α2
E307
α1
α3
α6
Y310
I309
E307
L308
I306
α6
L308
I309
I306
E307
α6
Form I / Form II
Form I
Form II

e

	Area (Å^2)	Volume (Å^3)
Form I	573.1	425.8
Form II	523.6	314.5

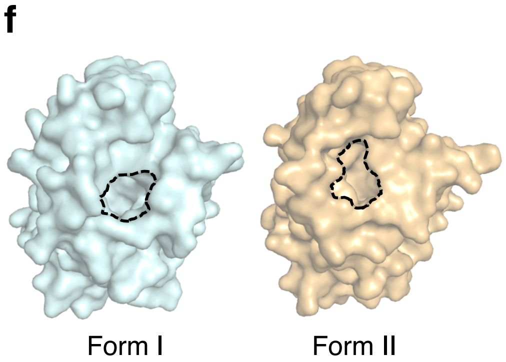

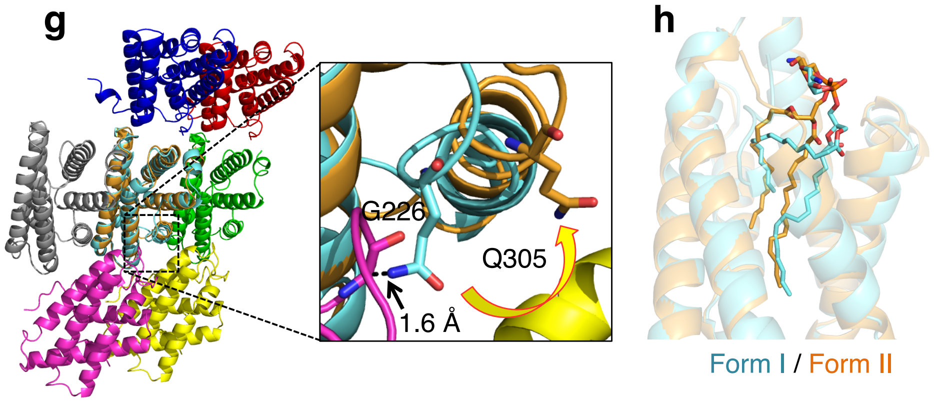

Supplementary Figure 3. Structural comparison of two forms of Gip1(146-310). (a) A Stereo view of Gip1(146-310; Form II) (PDB 5Z39). Overall structure is shown by a ribbon and stick model with the 2mFo-DFc electron density map contoured at 1.0 σ. Water molecules and a sodium ion are shown as red and purple balls, respectively. (b) R.m.s. deviations between Form I and Form II. The r.m.s. deviations (RMSD) of Cα atoms between Form I and Form II are plotted for each amino acid. (c) Structural difference between the two forms of Gip1 in the region between the α5 to α6 helices (a.a. 286-291) with a cartoon model (cyan; Form I, light orange; Form II). In Form I, the α6 helix is partially kinked by water molecules (red balls). On the other hand, α6 helix is tightly folded in Form II, which interacts with sodium ions (purple ball). Hydrogen bonds and coordination bonds are represented by yellow dashed lines. (d) Directional difference of Glu307 between Form I and Form II. The two forms (cyan; Form I, light

orange; Form II) are superimposed in cartoon representation with the side chains of Glu307 shown as stick models (left). Surface representation around the entrance of the cavity. The surface electrostatic potential is shown with the side chains depicted as stick models (Form I; middle, Form II, right). (e) Comparison of the size of the hydrophobic cavity. The data were calculated with the CASTp 3.0 server. (f) Rearrangement of cavity entrance. The structures of the two forms are shown in surface representation from the same viewpoint (Form I; left, Form II; right). The entrance of the cavity is surrounded by a dashed line. (g) Investigation of the molecular packing in two crystals. Symmetry-related molecules of Gip1 (Form II) are shown in various colours other than cyan. Gip1 (Form I; cyan) is superimposed on the central Gip1 (Form II) on the left. The contact region between the α6 helix and a symmetry-related molecule is surrounded by the dashed square. The right figure shows a close-up view of the contact region. The residues shown as stick models represent clashes with an adjacent molecule. For example, the distance between Gln305 of Form I (cyan) and Gly226 of the symmetry-related Form II (magenta) is too close (1.6 Å). (h) Structural comparison of accommodated lipids. The structures of the two forms are superimposed in cartoon representation with the accommodated phospholipids shown as stick models (Form I; cyan, Form II; light orange).

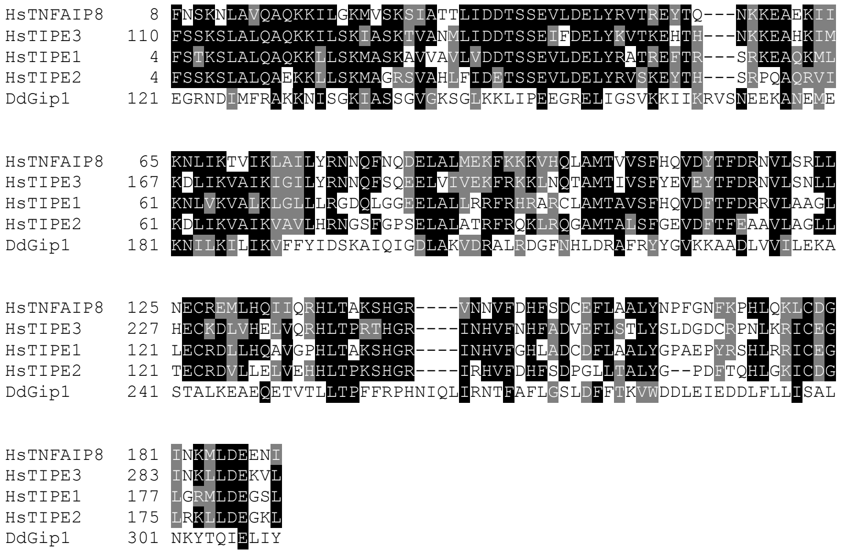

Supplementary Figure 4. Sequence comparison of Gip1 and TNFAIP8 family proteins. Sequence alignment of Gip1 and human TNFAIP8 family proteins. Alignment was generated by CLUSTALW[1]. Identical residues and similar residues are highlighted in black and grey, respectively. The alignment scores between Gip1 and TNFAIP8 (CAG33418.1), TIPE1 (sp|Q8WVP5.2), TIPE2 (sp|Q6P589.1), and TIPE3 (NP_997264.2) are 13.7, 14.5, 14.7, and 14.0, respectively. Hs, *Homo sapiens*; Dd, *Dictyostelium discoideum*.

a

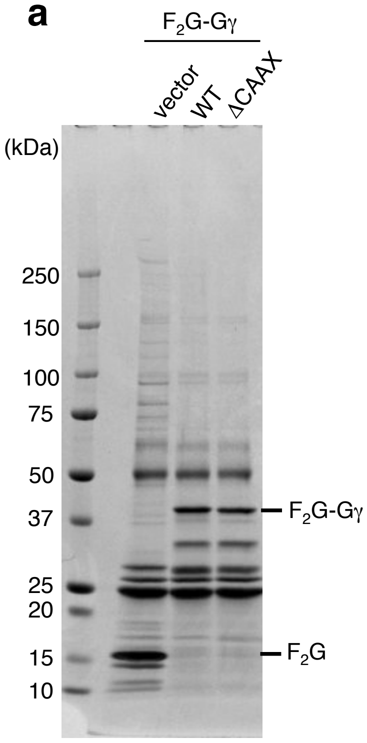

CBB staining

b

Gγ(WT)

MSESQLKKVL KENETLKAQL EKSTTILKVS EACESLQDYC
TKTSDPFIPG WSGENEWTKP LKGNGCSVL

Score	Expect	Peptide	
13	0.046	E.NEWTKPLKGNGC.-	+ GG+Met (C)
13	0.045	E.WTKPLKGNGC.-	GG+Met (C)
1	0.71	E.WTKPLKGNGC.-	+ GG+Met (C)
5	0.33	E.WTKPLKGNGC.-	+ GG+Met (C)
2	0.67	E.WTKPLKGNGC.-	+ GG+Met (C)
2	0.69	E.WTKPLKGNGC.-	+ GG+Met (C)

Gγ(ΔCAAX)

MSESQLKKVL KENETLKAQL EKSTTILKVS EACESLQDYC
TKTSDPFIPG WSGENEWTKP LKGNG

Score	Expect	Peptide
26	0.0028	E.NEWTKPLKGNG.-
11	0.089	E.NEWTKPLKGNG.-
13	0.052	E.WTKPLKGNG.-
12	0.067	E.WTKPLKGNG.-
14	0.038	E.WTKPLKGNG.-
12	0.063	E.WTKPLKGNG.-
24	0.0043	E.WTKPLKGNG.-
12	0.067	E.WTKPLKGNG.-
8	0.14	E.WTKPLKGNG.-
35	0.00033	E.WTKPLKGNG.-
22	0.0062	E.WTKPLKGNG.-

c

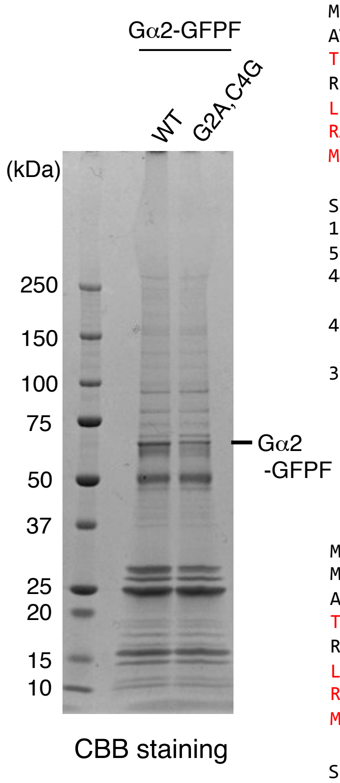

d

Gα2(WT)

```
MGICASSMEG EKTNTDINLS IEKERKKKHN EVKLLLLGAG ESGKSTISKQ
MKIIHQSGYS NEERKEFKPI ITRNILDNMR VLLDQMGRLG MTIDPSNSDA
AVMIKELTSL QASIVTDCWG ELNEDQGKKI KALWTDPGVK QAMRRANEFS
TLPDSAPYFF DSIDRMTSPV YIPTDQDILH TRVMTRGVHE TNFEIGKIKF
RLVDVGGQRS ERKKWLSCFD DVTAVVFCVA LSEYDLLLYE DNSTNRMLES
LRVFSDVCNS WFVNTPIILF LNKSDLFREK IKHVDLSETF PEYKGGRDYE
RASNYIKERF WQINKTEQKA IYSHITCATD TNNIRVVFEA VKDIIFTQCV
MKAGLY
```

Score	Expect	Peptide
16	0.023	M.GICASSMEGEK.T + Oxidation (M)
50	1.1e-005	M.GICASSMEGEK.T + Carbamidomethyl (C);
44	4e-005	M.GICASSMEGEK.T + Carbamidomethyl (C) Oxidation (M)
40	0.0001	M.GICASSMEGEK.T + Carbamidomethyl (C); Myristoyl (N-term G)
38	0.00017	M.GICASSMEGEK.T + Carbamidomethyl (C); Myristoyl (N-term G); Oxidation (M)

Gα2(G2A,C4G)

```
MAIGASSMEG EKTNTDINLS IEKERKKKHN EVKLLLLGAG ESGKSTISKQ
MKIIHQSGYS NEERKEFKPI ITRNILDNMR VLLDQMGRLG MTIDPSNSDA
AVMIKELTSL QASIVTDCWG ELNEDQGKKI KALWTDPGVK QAMRRANEFS
TLPDSAPYFF DSIDRMTSPV YIPTDQDILH TRVMTRGVHE TNFEIGKIKF
RLVDVGGQRS ERKKWLSCFD DVTAVVFCVA LSEYDLLLYE DNSTNRMLES
LRVFSDVCNS WFVNTPIILF LNKSDLFREK IKHVDLSETF PEYKGGRDYE
RASNYIKERF WQINKTEQKA IYSHITCATD TNNIRVVFEA VKDIIFTQCV
MKAGLY
```

Score	Expect	Peptide
29	0.0013	M.AIGASSMEGEK.T
66	2.5e-007	M.AIGASSMEGEK.T
44	4e-005	M.GICASSMEGEK.T + Carbamidomethyl (C); Oxidation (M)
40	0.0001	M.GICASSMEGEK.T + Carbamidomethyl (C); Myristoyl (N-term G)
38	0.00017	M.GICASSMEGEK.T + Carbamidomethyl (C); Myristoyl (N-term G); Oxidation (M)

Supplementary Figure 5. Mass spectrometric identification of prenyl modification of Gγ and myristoylation of Gα2. (a) Preparation for mass spectrometric analysis of Gγ. The indicated proteins were purified by anti-Flag beads and separated. (b) Identification of chemical modifications on Gγ. The identified peptides of Gγ(WT) and Gγ(ΔCAAX) are shown in red. The geranylgeranylated and methylated peptide was identified only in Gγ(WT) and not in Gγ(ΔCAAX). (c) Preparation for mass spectrometric analysis of Gα2. (d) Identification of chemical modifications on Gα2.

The identified peptides of Gα2(WT) and Gα2(G2A,C4G) are shown in red. The myristoylated peptide was identified only in Gα2(WT) and not in Gα2(G2A,C4G).

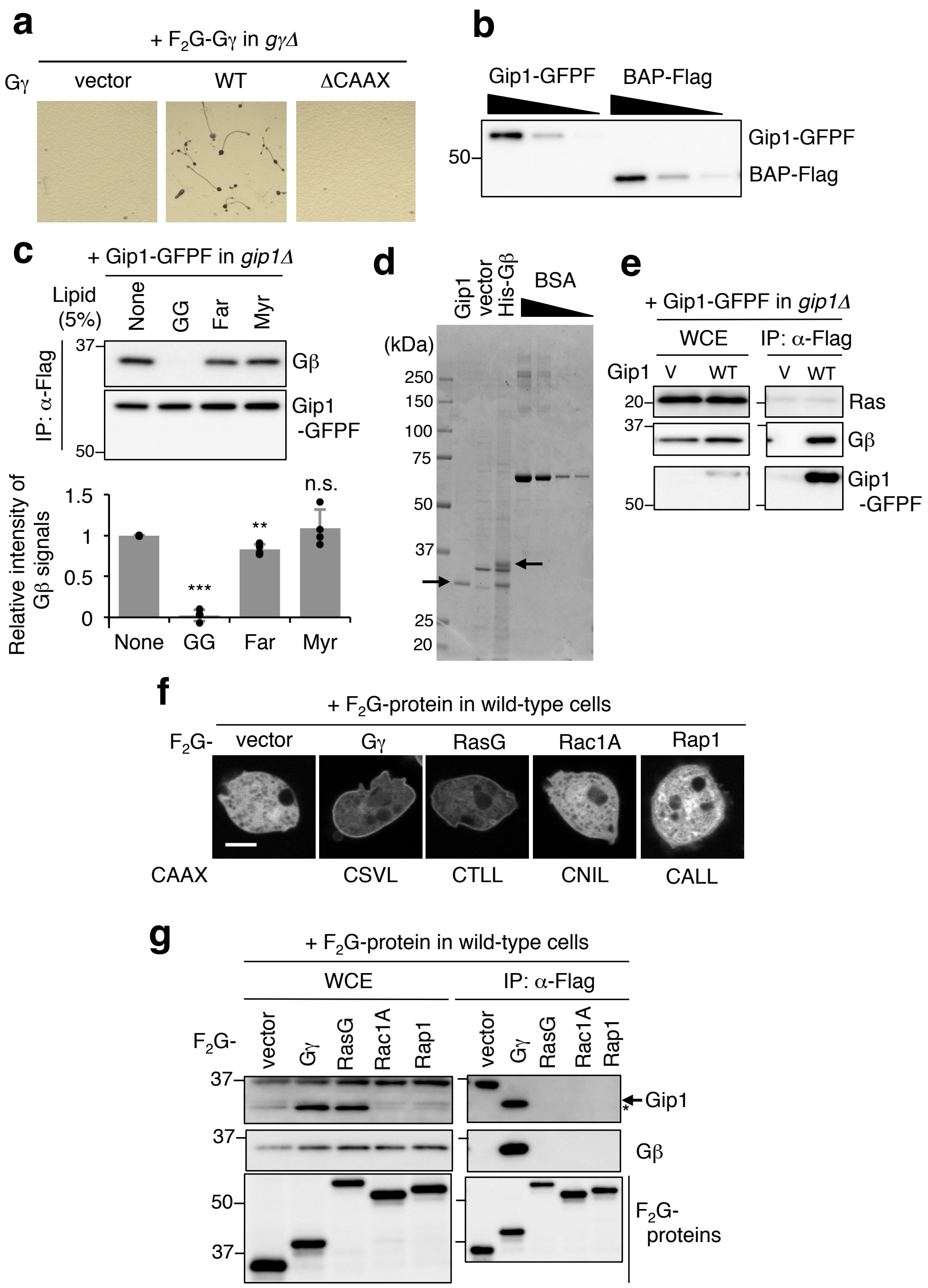

Supplementary Figure 6. Complex formation via the geranylgeranyl modification on Gγ. (a) Developmental phenotypes of Gγ mutant cells upon starvation. Flag-Flag-GFP (F_2G) tag alone (vector) or F_2G-tagged Gγ(WT) or Gγ(ΔCAAX) in *gγΔ* cells. Cells were developed on non-nutrient agar overnight and observed to have formed fruiting bodies. (b) Quantification of the amount of Gip1-GFPF bound to anti-Flag

beads used for the competitive assay shown in Fig. 3e. For this purpose, 0.5, 0.17, and 0.05% of a sample containing Gip1-GFPF were immunoblotted with an anti-Flag antibody together with 1, 0.33, and 0.1 ng of BAP-Flag as a standard. (c) Competitive dissociation of G proteins from Gip1 by the same concentration (100 μM) of geranylgeranyl pyrophosphate (GG), farnesyl pyrophosphate (Far), and myristic acid (Myr). The data were normalized relative to the band intensities without lipid and represent the mean ± SD of four independent experiments ($n = 4$, $^{**}P < 0.01$, $^{***}P < 0.001$ versus 0% lipid, two-tailed unpaired Student's *t*-test). (d) Quantification of His-Gβ and full-length Gip1. Both proteins were bacterially expressed and purified, as shown by the arrows. These proteins, along with 500, 200, 50, and 20 ng of BSA, were separated on a polyacrylamide gel and visualized by Coomassie Brilliant Blue. (e) Binding specificity of Gip1. Gip1-GFPF proteins were pulled down, followed by immunoblotting with anti-Ras, anti-Gβ, and anti-Flag antibodies. (f) Subcellular localization of proteins with a CAAX motif. The indicated proteins with GFP were observed by confocal microscopy. The amino acid sequences of each CAAX motif are shown under micrographs. (g) Binding of Gip1 to proteins with a CAAX motif. F_2G tagged proteins were pulled down, followed by immunoblotting with anti-Gip1, anti-Gβ, and anti-Flag antibodies.

a

	Selected with CASTp	Substituted to tryptophan	Residues within 4 Å from the phospholipid (Å)
	I152	○	3.8
α1	P153		3.6
α1	E155		3.6
α1	G156		3.3
α1	L159	○	3.7
α1	I160	○	3.8
α1	V163	○	3.6
α1	I166	○	
α2	I183	○	
α2	I186	○	3.9
α2	L187	○	3.7
α2	V190	○	
α2	F191		3.6
α3	L204	○	
α3	V207	○	3.9
α3	D208		
α3	L211	○	3.6
α3	F215		
α3	L218	○	3.8

	Selected with CASTp	Substituted to tryptophan	Residues within 4 Å from the phospholipid (Å)
α4	L244	○	
α4	T251		
α4	L254	○	
α5	T270		
α5	F271		3.5
α5	L274	○	
α5	G275		
α5	F279		3.9
α5	F280		3.9
α5	V283	○	
α5	W284		
α6	L293	○	4.0
α6	L296	○	3.8
α6	I297	○	
α6	L300	○	
	Y303		3.3
	I306	○	
	E307		
	L308	○	3.3
	I309	○	3.4
	Y310		3.1

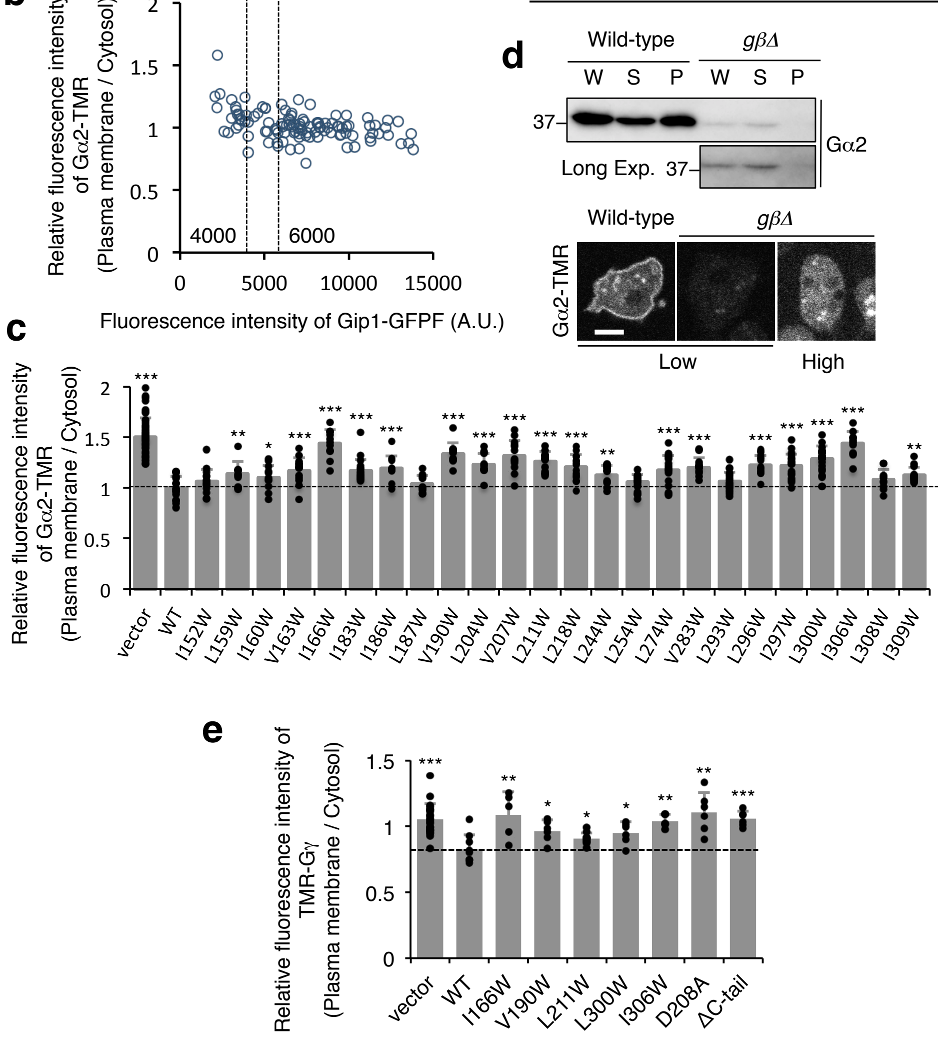

Supplementary Figure 7. Tryptophan mutagenesis scan inside the cavity. (a) Comprehensive selection of residues inside the cavity for tryptophan mutagenesis. Forty residues were identified by CASTp as composing the surface inside the cavity. Twenty-four residues were selected for mutagenesis. Twenty-two residues surrounding a phospholipid within 4 Å are included in the list, with the closest distance from the phospholipid. (b) Representative scatter plot of the fluorescence intensities of Gip1-GFPF and Gα2-TMR. The data plots shown are quantified from wild-type cells. For further validation, data plots between Gip1-GFP intensities of 4,000 and 6,000 (shown inside the dashed lines) are used. (c) Effects of tryptophan mutagenesis scan. The data represent the mean ± SD ($n \geq 10$ cells, *$P < 0.05$, **$P < 0.01$, ***$P < 0.001$ versus wild type, two-tailed unpaired Student's *t*-test). (d) Gα2 localization in wild-type or *gβΔ* cells. The indicated cells were fractionated as in Fig. 4. Protein samples equivalent to 1.5×10^5 cells were immunoblotted with an anti-Gα2 antibody (upper panel). Confocal micrographs of Gα2-TMR are shown in a wild-type or *gβΔ* cell. For a *gβΔ* cell, a representative image at high-laser-power values is shown due to its weak signals. (e) Effects of the tryptophan mutagenesis scan, D208A, and ΔC-tail, assessed by TMR-Gγ instead of Gα2-TMR. The data represent the mean ± SD ($n \geq 5$ cells, *$P < 0.05$, **$P < 0.01$, ***$P < 0.001$ versus wild type, two-tailed unpaired Student's *t*-test).

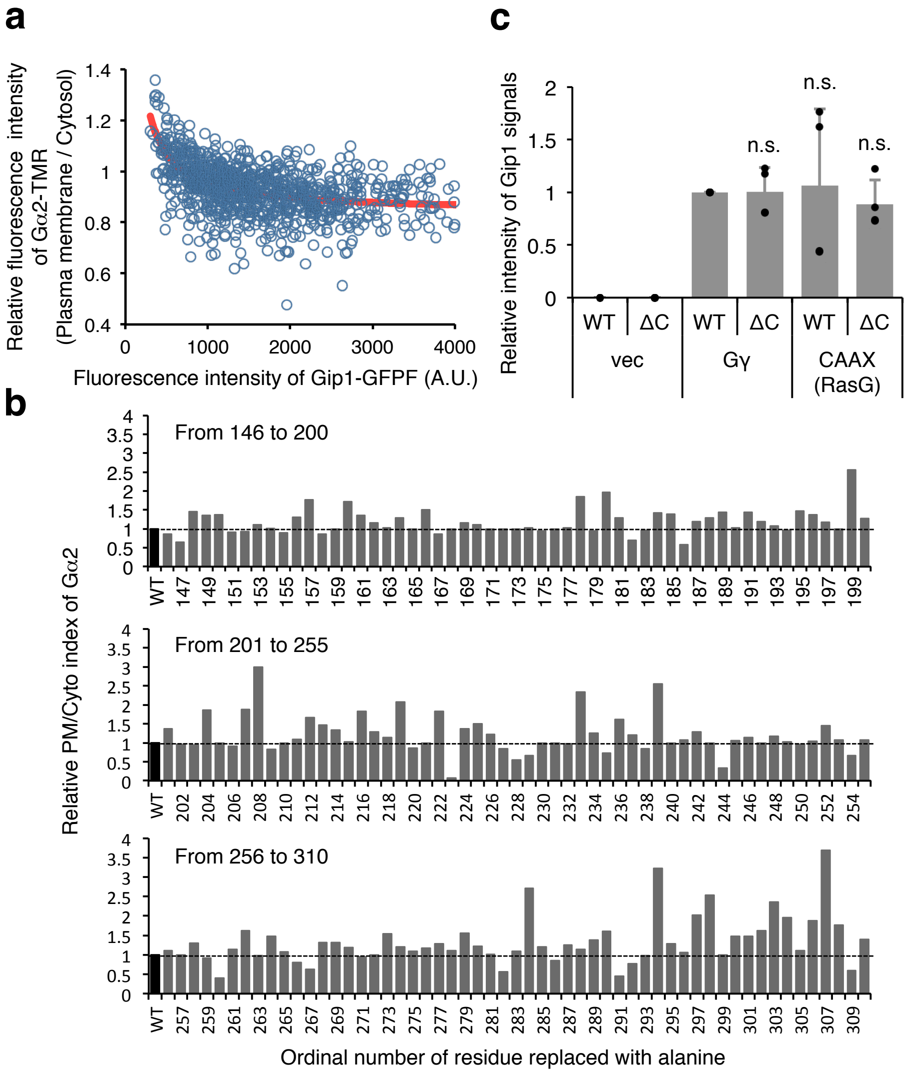

Supplementary Figure 8. Comprehensive alanine mutagenesis. (a) Representative scatter plot of the fluorescence intensities of Gip1-GFPF and Gα2-Halo. The data plots shown are the quantified values from wild-type cells. The data plots were fitted with a hyperbolic curve ($y = A/x + C$). A is used as a PM/Cyto index. (b) Effects of the alanine mutagenesis scan. The data are the obtained A values ($n \geq 50$ cells). (c) Quantification of the in vitro binding ability of purified Gip1 to prenylated proteins shown in Fig. 6d. Purified Gip1 was wild type (a.a. 1-310) and ΔC-tail (a.a. 1-303). Prenylated proteins were F_2G, F_2G-Gγ, and F_2G-RasG(178-189). The results are normalized relative to the

band intensities of wild-type Gip1 bound to F_2G-Gγ and represent the mean ± SD of four independent experiments (*n* = 4, two-tailed unpaired Student's *t*-test versus Gip1(WT) to F_2G-Gγ).

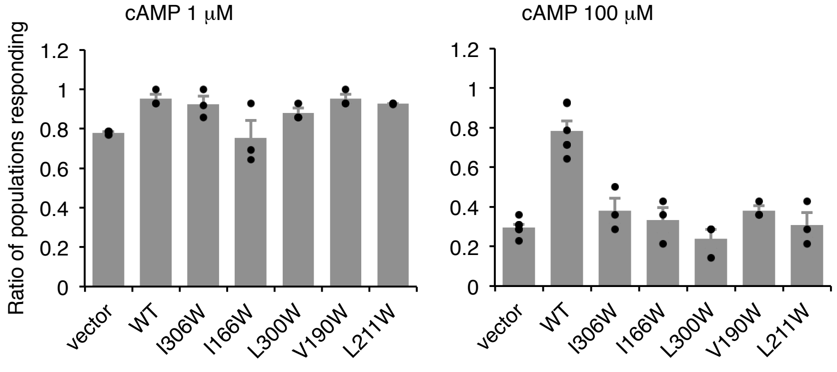

Supplementary Figure 9. Chemotactic efficiency of tryptophan-substituted cells.

Chemotactic response to 1 and 100 μM cAMP. The mutant cells with Trp mutations in the hydrophobic cavity were analysed as in Fig. 7c. The data represent the mean ± SEM of at least three independent experiments (*n* = 4 for vector (cAMP 100 μM) and WT (cAMP 100 μM), *n* = 3 for the other experiments).

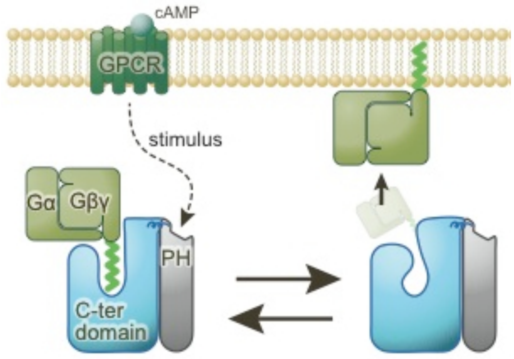

Supplementary Figure 10. Schematic model of G protein shuttling. Structure-based model of Gip1-mediated G protein shuttling. In the resting state, G proteins are not permanently anchored on the plasma membrane. Some detach from the membrane, resulting in cytosolic sequestration by interaction with Gip1. The structural basis of the complex formation is the interaction of the hydrophobic cavity of Gip1 and the lipid modification of G proteins. Chemoattractant signalling enforces the dissociation of G proteins from Gip1 by a plausible conformational change of the hydrophobic cavity. The N-terminal PH domain of Gip1 can modulate the configuration of the cavity upon cAMP stimulation.

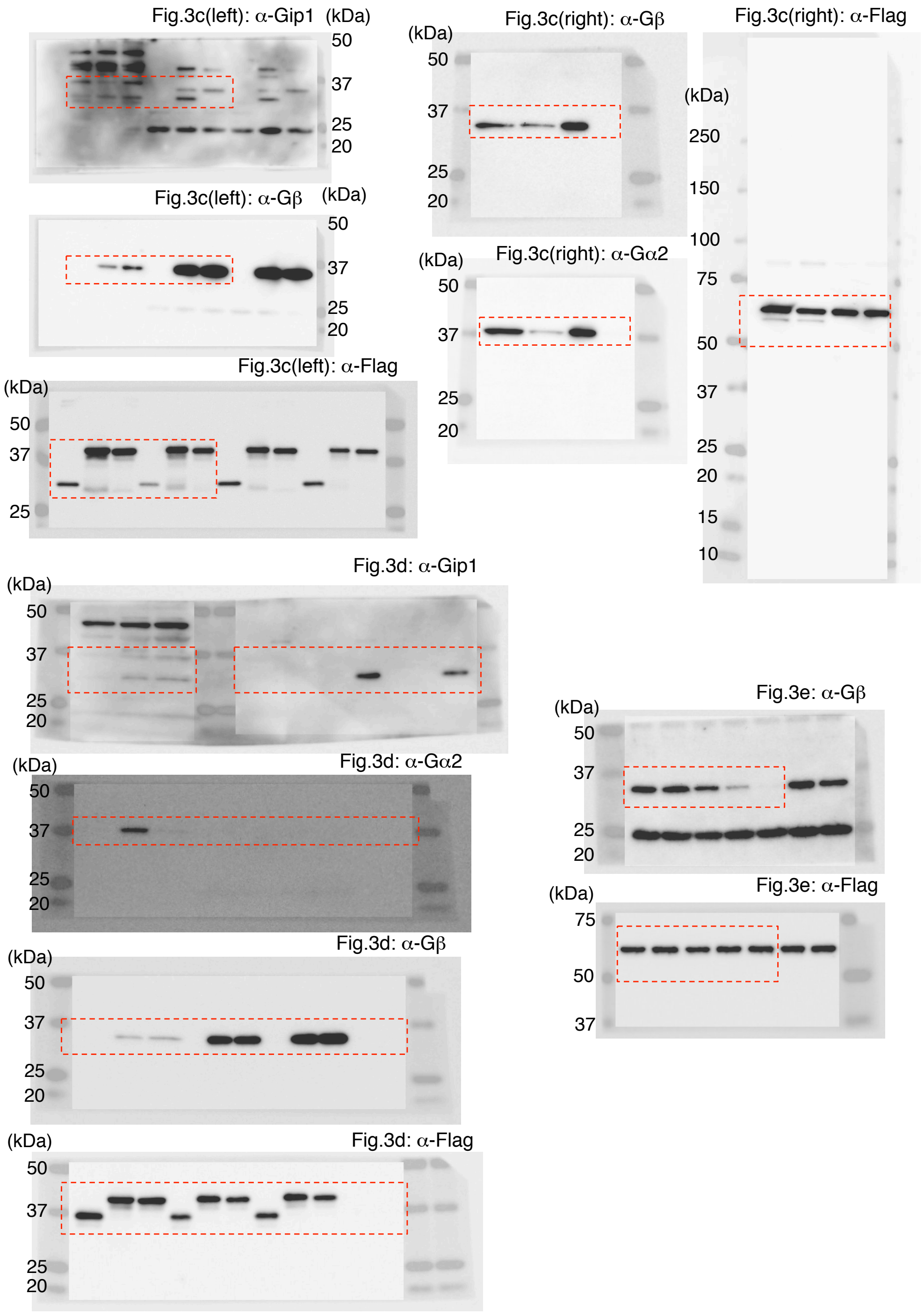
Fig.3c(left): α-Gip1
(kDa)
50
37
25
20
Fig.3c(left): α-Gβ
(kDa)
50
37
25
20
Fig.3c(left): α-Flag
(kDa)
50
37
25
Fig.3c(right): α-Gβ
(kDa)
50
37
25
20
Fig.3c(right): α-Gα2
(kDa)
50
37
25
20
Fig.3c(right): α-Flag
(kDa)
250
150
100
75
50
37
25
20
15
10
Fig.3d: α-Gip1
(kDa)
50
37
25
20
Fig.3d: α-Gα2
(kDa)
50
37
25
20
Fig.3d: α-Gβ
(kDa)
50
37
25
20
Fig.3d: α-Flag
(kDa)
50
37
25
20
Fig.3e: α-Gβ
(kDa)
50
37
25
20
Fig.3e: α-Flag
(kDa)
75
50
37

Fig.4a: α-Gip1

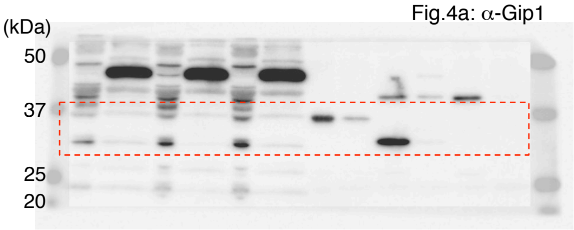

Fig.4a: α-Gβ

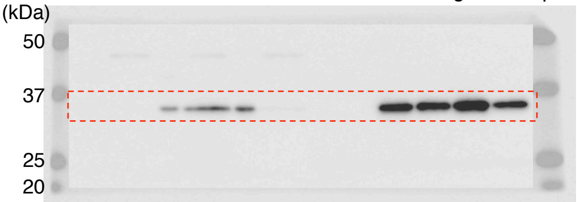

Fig.4a: α-Flag

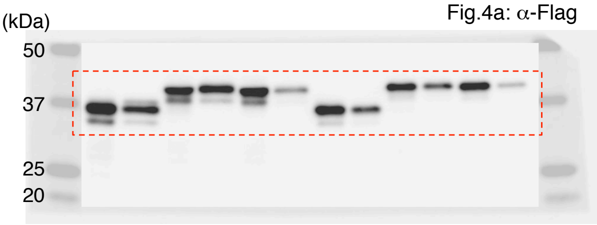

Fig.4b: α-Gβ

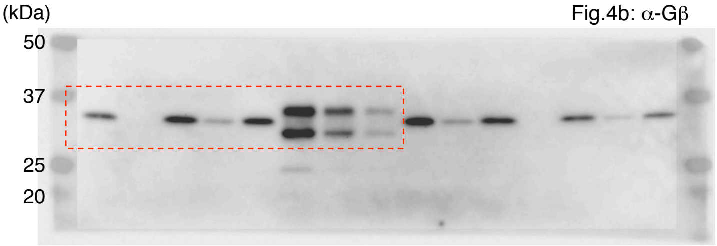

Fig.4b: α-Gip1

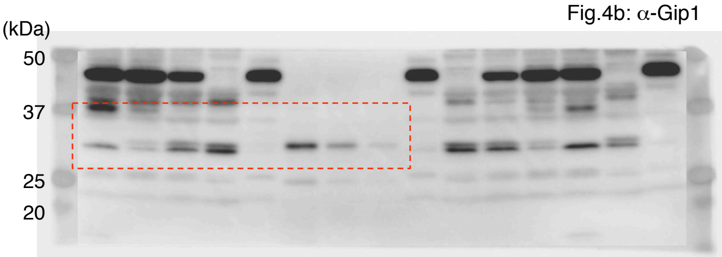

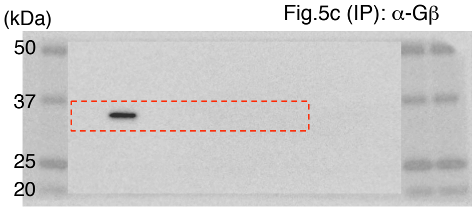

Fig.5c (IP): α-Gβ

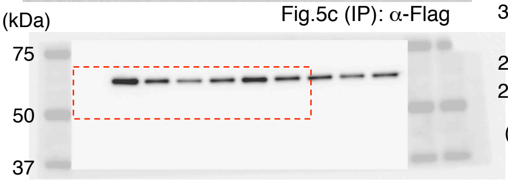

Fig.5c (IP): α-Flag

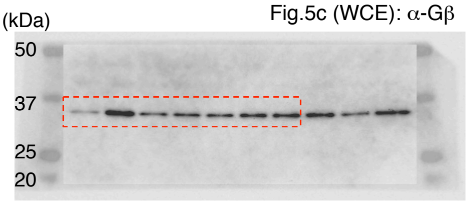

Fig.5c (WCE): α-Gβ

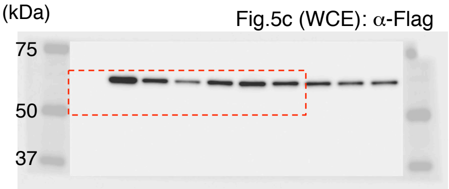

Fig.5c (WCE): α-Flag

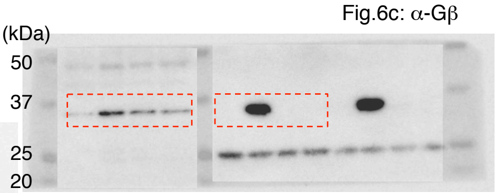

Fig.6c: α-Gβ

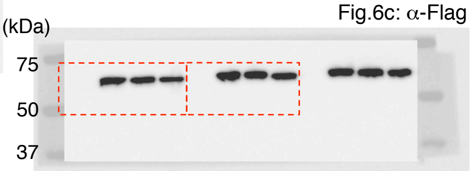

Fig.6c: α-Flag

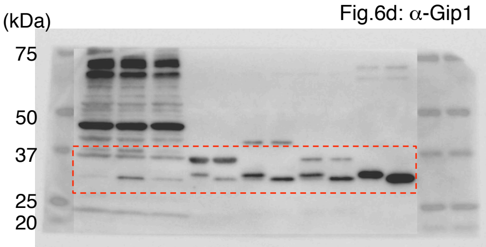

Fig.6d: α-Gip1

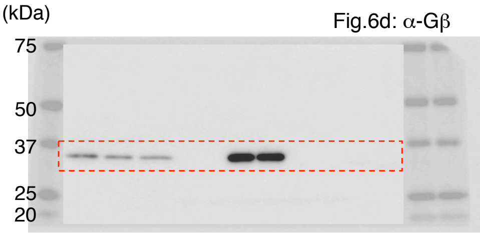

Fig.6d: α-Gβ

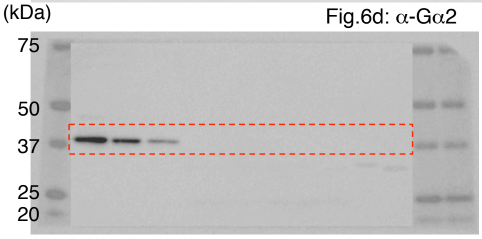

Fig.6d: α-Gα2

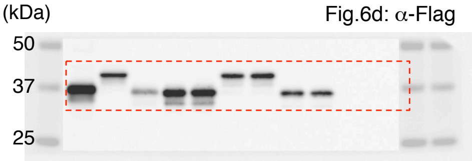

Fig.6d: α-Flag

Supplementary Fig.6b: α-Flag

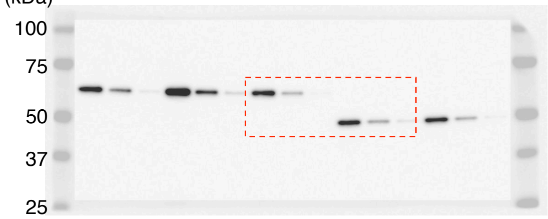

Supplementary Fig.6c: α-Gβ

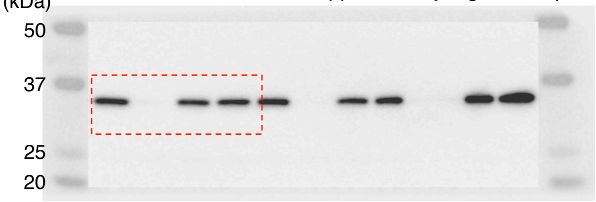

Supplementary Fig.6c: α-Flag

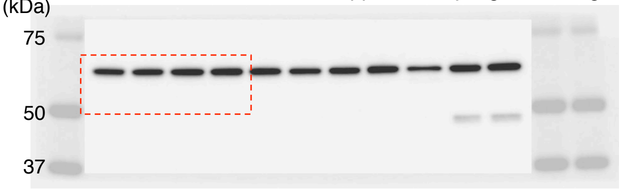

Supplementary Fig.6e: α-Ras

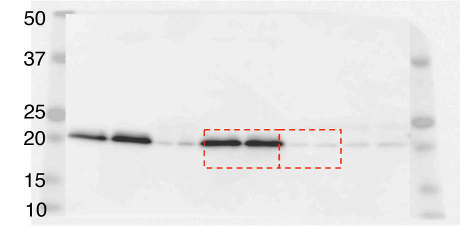

Supplementary Fig.6e: α-Gβ

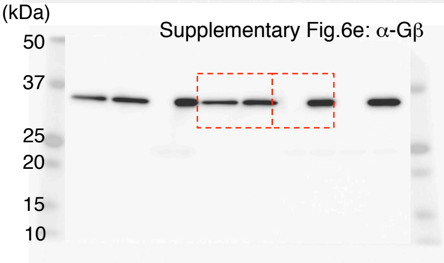

Supplementary Fig.6e: α-Flag

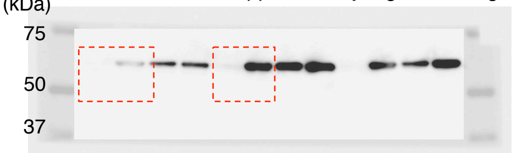

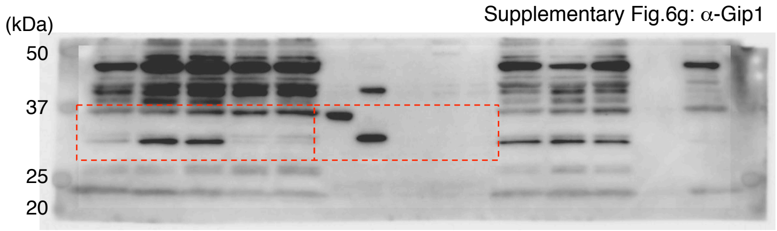

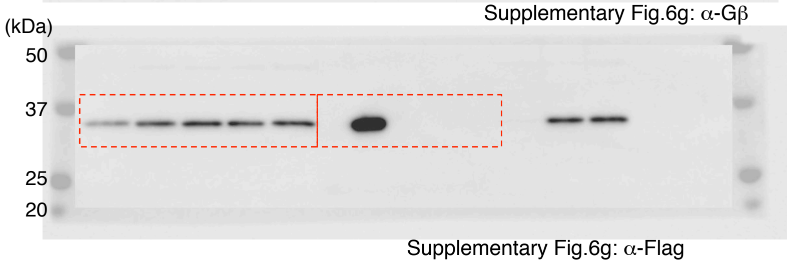

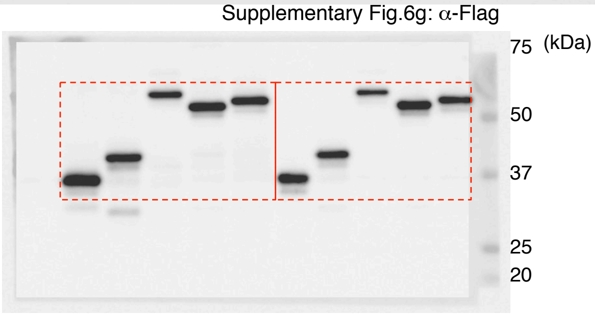

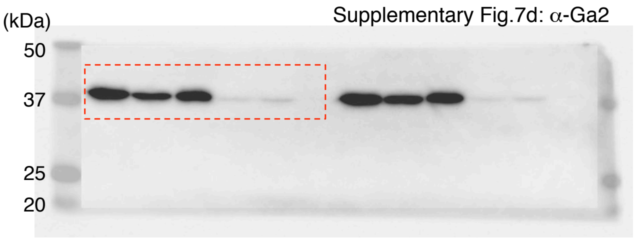

Supplementary Figure 11. Uncropped immunoblots.

Supplementary Table 1. Data collection and refinement statistics

	Gip1(Form I)	Gip1(Form II)
**Data collection**		
Space group	$P2_12_12_1$	$P2_12_12_1$
Cell dimensions		
a, *b*, *c* (Å)	33.32, 44.14, 96.64	33.47, 43.62, 101.69
Resolution (Å)	50.00 – 1.95 (1.98 – 1.95)*	43.62 – 2.74 (2.87 – 2.74)
R_{merge} (%)‡	9.0 (35.3)	11.9 (59.3)
$I / \sigma(I)$#	17.7 (2.9)	18.9 (4.8)
Completeness (%)	98.3 (66.2)	100.0 (100.0)
Redundancy	3.8 (3.2)	13.5 (13.9)
Total reflections	41,315	57,793
Unique reflections	10,906	4,277
**Refinement**		
Resolution (Å)	48.32 – 1.95	43.62 – 2.74
No. reflections	10,864	4,242
R_{work} / R_{free}	0.18/0.23	0.21/0.27
No. atoms		
Protein	1,373	1,356
Ligand/ion	53	53
Water	110	7
B-factors		
Protein	18.2	42.0
Ligand/ion	28.1	50.8
Water	23.8	34.0
R.m.s. deviations		
Bond lengths (Å)	0.003	0.002
Bond angles (°)	0.602	0.456

Each data set was collected from a single crystal.

*Values in parentheses are for highest-resolution shell.

‡$R_{merge} = \Sigma_{hkl} |I(hkl) - \langle I(hkl)\rangle| / \Sigma_{hkl} I(hkl)$, where $\langle I(hkl)\rangle$ is the mean of the symmetry-equivalent reflections of $I(hkl)$.

#$I/\sigma(I) = \langle I\rangle / \langle\sigma(I)\rangle$ for Form I (processed with HKL2000) and $= \langle I / \sigma(I)\rangle$ for Form II (processed with XDS).

Supplementary Table 2. Structural comparison of lipid-binding proteins based on PDB structures

	PDB ID	Fold /Binding site	Ligand in PDB	Lipid [*1]	Surface ($Å^2$)	Volume ($Å^3$)	Ref.
Gip1 (Form I)	5Z1N	cylinder-like /cavity	PE/PG	-	573.1	425.8	this study
Gip1 (Form II)	5Z39	cylinder-like /cavity	PE/PG	-	523.6	314.5	this study
RhoGDI1	1RHO	Ig-like /cavity	Free	-	128.3	99.2	2
RhoGDI1	1DOA	Ig-like /cavity	Cdc42 (GDP)	GG	329.9	218.2	3
RhoGDI1	1HH4	Ig-like /cavity	Rac1 (GDP)	GG	250.4	115.0	4
RhoGDI1	4F38	Ig-like /cavity	RhoA (GppNHp)	GG	446.2	250.4	5
RhoGDI2	1DS6	Ig-like /cavity	Rac2 (GDP)	-	239.0	171.8	6
PDEδ	1KSG	Ig-like /cavity	Arl2 (GTP)	-	58.9	31.8	7
PDEδ	3T5G	Ig-like /cavity	RheB (GDP)	Far	383.4	207.2	8
PDEδ	4JHP	Ig-like /cavity	RPGR RCC1 -like domain	-	422.4	214.1	9
PDEδ	5TAR	Ig-like /cavity	KRas4B (GDP)	Far	464.4	309.3	10
PDEδ	5TB5	Ig-like /cavity	KRas4B (GDP)	Far	498.0	311.4	10
PDEδ	5F2U	Ig-like /cavity	INPP5E peptide	Far	458.6	299.5	11
PDEδ	5T4X	Ig-like /cavity	Free	-	411.2	310.5	12
UNC119a	3GQQ	Ig-like /cavity	Free	-	515.9	344.1	13
UNC119a	3RBQ	Ig-like /cavity	Gαt mimic peptide	Lau	459.0	307.9	13
UNC119a	4GOJ	Ig-like /cavity	Arl3 (GppNHp)	-	442.5	212.3	14
UNC119a	4GOK	Ig-like /cavity	Arl2 (GppNHp)	-	N.D. [*2]	N.D. [*2]	14
UNC119a	5L7K	Ig-like /cavity	NPHP3 peptide	Myr	283.4	133.3	15
RabGDI	1GND	REP[*3]-like /pocket	Free	-	29.5	6.2	16

RabGDI	1LV0	REP[*3]-like /pocket	GG-peptide	GG[*4]	24.6	4.2	17
RabGDI	1UKV	REP[*3]-like /pocket	Ypt1 (GDP)	GG (×1)	360.0	336.9	18
RabGDI	2BCG	REP[*3]-like /pocket	Ypt1 (GDP)	GG (×2)	366.8	364.9	19
TIPE2	3F4M	cylinder-like /cavity	-	-	N.D. [*2]	N.D. [*2]	20
TIPE3	4Q9V	cylinder-like /cavity	-	-	604.4	385.3	21
Tnfaip8	5JXD	cylinder /cavity	PE	-	518.6	349.8	22

The size of the cavity was calculated with the CASTp 3.0 server.

*1) Lipid indicates the lipid modification of ligands in PDB structures. GG, Far, Lau, and Myr are geranylgeranyl, farnesyl, lauryl, and myristoyl groups, respectively.

*2) The hydrophobic cavity was not precisely determined due to partially lacking the structural information.

*3) REP is Rab escort protein.

*4) This geranylgeranyl peptide binds to different sites from those in 1UKV and 2BCG.

Supplementary Table 3. List of all used primers in this study

**Name**	**Plasmid**	**Direction**	**Sequence (5' to 3')**	**Comments**
TM54	pE-6HisSUMO-3C -Gip1(1-310)	F	GAACAGATTGGAGGTCTGGAAGTTCTGTTCC AGGGGCCC	
YK621	pE-6HisSUMO-3C -Gip1(1-310)	R	ATTCGGATCCTCTAGTTAATAAATCAATTCA ATTTGAGTATATTTG	
TM55	pE-8HisSUMO-3C -Gip1(1-310)	R	ACCCATGGTATATCTCCTTCTTAAAGT	
TM57	pE-8HisSUMO-3C -Gip1(1-310)	F	CACCACCATCACCATCATCATCACGGGTCCC TGCAGGACTCAGAA	
TM109	pE-8HisSUMO-3C -Gip1(146-310)	F	TTCCAGGGGCCCCTGAGTGGTTTAAAGAAAT TGATTCCA	
TM110	pE-8HisSUMO-3C -Gip1(146-310)	R	ACGGAGCTCGAATTCTTAATAAATCAATTCA ATTTGAGTATATTTGTT	
TM211	pE-8HisSUMO-3C -Gip1(146-303)	R	ACGGAGCTCGAATTCTTA ATATTTGTTAAGAGCACTAATTAATAAGAAT A	
TM212	pE-8HisSUMO-3C -Gip1(1-310; DC-tail)	F	TTCCAGGGGCCCCTG ATGGAGGCAATTACAATTGAAA	
YK383	pTX-F_2G-Gg (DCAAX)	F	AGGTGGTGGAGGATCCGAATCACAATTAAA AAAAG	
YK819	pTX-F_2G-Gg (DCAAX)	R	ATCGTCTAGACTCGAGTTATCCATTTCCTTT GAGTGGTTTAGTCC	
YK899	pTX-F_2G-CAAX(RasG)	F	AGGTGGTGGAGGATCCAAGAAGAAGAGACC ATTAAAAGCTTGTACTCTTTTATAACTCGAG TCTAGACGAT	
YK900	pTX-F_2G-CAAX(RasG)	R	ATCGTCTAGACTCGAGTTATAAAAGAGTAC AAGCTTTTAATGGTCTCTTCTTCTTGGATCCT CCACCACCT	
YK374	pTX-F_2G-RasG	F	CCAGATCTGGTGGTGGAGGTGGTGGAGGAT CCATGACAGAATACAAATTAGTTA	
YK361	pTX-F_2G-RasG	R	TCTAGATTATAAAAGAGTACAAGCTTTTAAT GGTCTC	
YK491	pTX-F_2G-Rac1A	F	AGGTGGTGGAGGATCCATGCAAGCAATTAA ATGTGTCGTTGTCGGTGATGG	
YK492	pTX-F_2G-Rac1A	R	ATCGTCTAGACTCGAGTTATAAAATGTTGCA ACCACCTGAAC	
YK485	pTX-F_2G-Rap1	F	AGGTGGTGGAGGATCCATGCCTCTTAGAGA ATTCAAAATCGTCG	
YK486	pTX-F_2G-Rap1	R	ATCGTCTAGACTCGAGTTACAATAAAGCAC ATTTTGATTTAGC	
YK459	pJK1-Gip1(DC-tai l)-GFPF	F	AATAAAAATCAGATCAAATAAAAATGGAGG CAATTACAATTGAAATTAATC	
YK927	pJK1-Gip1(DC-tai l)-GFPF	R	CACCACCTCCCTCGAGATATTTGTTAAGAGC ACTAATTAATAAG	
YK874	pJK1-Ga2(G2A, C4G)-GFPF	F	AATAAAAATCAGATCAAATAAAAATGGCTA TTGGTGCATCATCAATGG	
YK411	pJK1-Ga2(G2A, C4G)-GFPF	R	TACCTCTAGCAGATCTTAAGAATATAAACCA GCTTTCATAAC	
**Ala/Trp mutaegnesis**				
**Name**	**pJK1-Gip1-G FPF**			
YK259 (Universal Gip1-F)		F	AATAAAAATCAGATCAAATAAAAATGGAGGC[illegible] TTACAATTGAAATTAATC	The 5'-end primer

YK460 (Universal Gip1-R)		R	CACCACCTCCCTCGAGATAAATCAATTCAATTTGAGTATATTTG	The 3'-end primer
Extension Gip1-R		R	CCAAATCATCCCAAACTTTAGTG	The 3' primer of the N termial fragment for E288A, I289A, E290A, D291A, D292A, L293A, F294A, L295A, L296A.
S146A-F	S146A	F	GTTGGTAAGGCTGGTTTAAAGAAATTG	
S146A-R	S146A	R	CAATTTCTTTAAACCAGCCTTACCAAC	
G147A-F	G147A	F	GTTGGTAAGAGTGCTTTAAAGAAATTG	
G147A-R	G147A	R	CAATTTCTTTAAAGCACTCTTACCAAC	
L148A-F	L148A	F	GTTGGTAAGAGTGGTGCAAAGAAATTG	
L148A-R	L148A	R	CAATTTCTTTGCACCACTCTTACCAAC	
K149A-F	K149A	F	GGTAAGAGTGGTTTAGCAAAATTGATTCCAGAAG	
K149A-R	K149A	R	CTTCTGGAATCAATTTTGCTAAACCACTCTTACC	
K150A-F	K150A	F	GGTAAGAGTGGTTTAAAGGCATTG	
K150A-R	K150A	R	CAATGCCTTTAAACCACTCTTACC	
L151A-F	L151A	F	GGTTTAAAGAAAGCAATTCCAGAAGAG	
L151A-R	L151A	R	CTCTTCTGGAATTGCTTTCTTTAAACC	
I152A-F	I152A	F	GGTTTAAAGAAATTGGCTCCAGAAGAG	
I152A-R	I152A	R	CTCTTCTGGAGCCAATTTCTTTAAACC	
P153A-F	P153A	F	GGTTTAAAGAAATTGATTGCAGAAGAG	
P153A-R	P153A	R	CTCTTCTGCAATCAATTTCTTTAAACC	
E154A-F	E154A	F	CCAGCAGAGGGTCGTGAATTG	
E154A-R	E154A	R	CAATTCACGACCCTCTGCTGG	
E155A-F	E155A	F	CCAGAAGCAGGTCGTGAATTG	
E155A-R	E155A	R	CAATTCACGACCTGCTTCTGG	
G156A-F	G156A	F	CCAGAAGAGGCTCGTGAATTG	
G156A-R	G156A	R	CAATTCACGAGCCTCTTCTGG	
R157A-F	R157A	F	CCAGAAGAGGGTGCTGAATTG	
R157A-R	R157A	R	CAATTCAGCACCCTCTTCTGG	
E158A-F	E158A	F	CCAGAAGAGGGTCGTGCATTG	
E158A-R	E158A	R	CAATGCACGACCCTCTTCTGG	
L159A-F	L159A	F	CGTGAAGCAATTGGATCAGTTAAAAAG	

L159A-R	L159A	R	CTTTTTAACTGATCCAATTGCTTCACG	
I160A-F	I160A	F	CGTGAATTGGCTGGATCAGTTAAAAAG	
I160A-R	I160A	R	CTTTTTAACTGATCCAGCCAATTCACG	
G161A-F	G161A	F	CGTGAATTGATTGCATCAGTTAAAAAG	
G161A-R	G161A	R	CTTTTTAACTGATGCAATCAATTCACG	
S162A-F	S162A	F	CGTGAATTGATTGGAGCAGTTAAAAAG	
S162A-R	S162A	R	CTTTTTAACTGCTCCAATCAATTCACG	
V163A-F	V163A	F	CGTGAATTGATTGGATCAGCTAAAAAG	
V163A-R	V163A	R	CTTTTTAGCTGATCCAATCAATTCACG	
K164A-F	K164A	F	CAGTTGCAAAGATCATTAAGAGAGTC	
K164A-R	K164A	R	GACTCTCTTAATGATCTTTGCAACTG	
K165A-F	K165A	F	CAGTTAAAGCAATCATTAAGAGAGTC	
K165A-R	K165A	R	GACTCTCTTAATGATTGCTTTAACTG	
I166A-F	I166A	F	CAGTTAAAAAGGCAATTAAGAGAGTC	
I166A-R	I166A	R	GACTCTCTTAATTGCCTTTTTAACTG	
I167A-F	I167A	F	CAGTTAAAAAGATCGCTAAGAGAGTC	
I167A-R	I167A	R	GACTCTCTTAGCGATCTTTTTAACTG	
K168A-F	K168A	F	CAGTTAAAAAGATCATTGCAAGAGTC	
K168A-R	K168A	R	GACTCTTGCAATGATCTTTTTAACTG	
R169A-F	R169A	F	CAGTTAAAAAGATCATTAAGGCAGTC	
R169A-R	R169A	R	GACTGCCTTAATGATCTTTTTAACTG	
V170A-F	V170A	F	CATTAAGAGAGCATCAAATGAAGAG	
V170A-R	V170A	R	CTCTTCATTTGATGCTCTCTTAATG	
S171A-F	S171A	F	CATTAAGAGAGTCGCAAATGAAGAG	
S171A-R	S171A	R	CTCTTCATTTGCGACTCTCTTAATG	
N172A-F	N172A	F	CATTAAGAGAGTCTCAGCTGAAGAG	
N172A-R	N172A	R	CTCTTCAGCTGAGACTCTCTTAATG	
E173A-F	E173A	F	GAGTCTCAAATGCAGAGAAAGC	
E173A-R	E173A	R	GCTTTCTCTGCATTTGAGACTC	
E174A-F	E174	F	GAGTCTCAAATGAAGCAAAAGC	
E174A-R	E174A	R	GCTTTTGCTTCATTTGAGACTC	
K175A-F	K175A	F	GAGTCTCAAATGAAGAGGCAGCAAATG	
K175A-R	K175A	R	CATTTGCTGCCTCTTCATTTGAGACTC	
N177A-F	N177A	F	GAGAAAGCAGCTGAAATGGAG	

N177A-R	N177A	R	CTCCATTTCAGCTGCTTTCTC	
E178A-F	E178A	F	GCAAATGCAATGGAGAAGAATATCC	
E178A-R	E178A	R	GGATATTCTTCTCCATTGCATTTGC	
M179A-F	M179A	F	GCAAATGAAGCAGAGAAGAATATCC	
M179A-R	M179A	R	GGATATTCTTCTCTGCTTCATTTGC	
E180A-F	E180A	F	GCAAATGAAATGGCAAAGAATATCC	
E180A-R	E180A	R	GGATATTCTTTGCCATTTCATTTGC	
K181A-F	K181A	F	GCAAATGAAATGGAGGCAAATATCCTAAAG ATTTTAATC	
K181A-R	K181A	R	GATTAAAATCTTTAGGATATTTGCCTCCATT TCATTTGC	
N182A-F	N182A	F	GGAGAAGGCTATCCTAAAGATTTTAATC	
N182A-R	N182A	R	GATTAAAATCTTTAGGATAGCCTTCTCC	
I183A-F	I183A	F	GGAGAAGAATGCACTAAAGATTTTAATC	
I183A-R	I183A	R	GATTAAAATCTTTAGTGCATTCTTCTCC	
L184A-F	L184A	F	GGAGAAGAATATCGCAAAGATTTTAATC	
L184A-R	L184A	R	GATTAAAATCTTTGCGATATTCTTCTCC	
K185A-F	K185A	F	GGAGAAGAATATCCTAGCAATTTTAATCAA AGTG	
K185A-R	K185A	R	CACTTTGATTAAAATTGCTAGGATATTCTTC TCC	
I186A-F	I186A	F	GGAGAAGAATATCCTAAAGGCTTTAATC	
I186A-R	I186A	R	GATTAAAGCCTTTAGGATATTCTTCTCC	
L187A-F	L187A	F	CCTAAAGATTGCAATCAAAGTG	
L187A-R	L187A	R	CACTTTGATTGCAATCTTTAGG	
I188A-F	I188A	F	CCTAAAGATTTTAGCAAAAGTG	
I188A-R	I188A	R	CACTTTTGCTAAAATCTTTAGG	
K189A-F	K189A	F	CCTAAAGATTTTAATCGCAGTGTTTTTCTAC ATTG	
K189A-R	K189A	R	CAATGTAGAAAAACACTGCGATTAAAATCT TTAGG	
V190A-F	V190A	F	GATTTTAATCAAAGCATTTTTCTAC	
V190A-R	V190A	R	GTAGAAAAATGCTTTGATTAAAATC	
F191A-F	F191A	F	GATTTTAATCAAAGTGGCTTTCTAC	
F191A-R	F191A	R	GTAGAAAGCCACTTTGATTAAAATC	
F192A-F	F192A	F	CAAAGTGTTTGCATACATTGATTC	
F192A-R	F192A	R	GAATCAATGTATGCAAACACTTTG	
Y193A-F	Y193A	F	CAAAGTGTTTTTCGCAATTGATTC	
Y193A-R	Y193A	R	GAATCAATTGCGAAAAACACTTTG	

I194A-F	I194A	F	CAAAGTGTTTTTCTACGCTGATTC	
I194A-R	I194A	R	GAATCAGCGTAGAAAAACACTTTG	
D195A-F	D195A	F	CTACATTGCTTCTAAAGCAATTC	
D195A-R	D195A	R	GAATTGCTTTAGAAGCAATGTAG	
S196A-F	S196A	F	CATTGATGCTAAAGCAATTC	
S196A-R	S196A	R	GAATTGCTTTAGCATCAATG	
K197A-F	K197A	F	CTACATTGATTCTGCAGCAATTC	
K197A-R	K197A	R	GAATTGCTGCAGAATCAATGTAG	
I199A-F	I199A	F	CTAAAGCAGCTCAAATTGGTG	
I199A-R	I199A	R	CACCAATTTGAGCTGCTTTAG	
Q200A-F	Q200A	F	CTAAAGCAATTGCAATTGGTG	
Q200A-R	Q200A	R	CACCAATTGCAATTGCTTTAG	
I201A-F	I201A	F	GCAATTCAAGCTGGTGATTTGGC	
I201A-R	I201A	R	GCCAAATCACCAGCTTGAATTGC	
G202A-F	G202A	F	GCAATTCAAATTGCTGATTTGGC	
G202A-R	G202A	R	GCCAAATCAGCAATTTGAATTGC	
D203A-F	D203A	F	GGTGCTTTGGCAAAGGTTG	
D203A-R	D203A	R	CAACCTTTGCCAAAGCACC	
L204A-F	L204A	F	GCAATTCAAATTGGTGATGCAGCAAAGG	
L204A-R	L204A	R	CCTTTGCTGCATCACCAATTTGAATTGC	
K206A-F	K206A	F	GGTGATTTGGCAGCAGTTGATAG	
K206A-R	K206A	R	CTATCAACTGCTGCCAAATCACC	
V207A-F	V207A	F	GATTTGGCAAAGGCTGATAGAGC	
V207A-R	V207A	R	GCTCTATCAGCCTTTGCCAAATC	
D208A-F	D208A	F	GGTTGCTAGAGCTTTACGTGACGG	
D208A-R	D208A	R	CCGTCACGTAAAGCTCTAGCAACC	
R209A-F	R209A	F	GGTTGATGCAGCTTTACGTGACGG	
R209A-R	R209A	R	CCGTCACGTAAAGCTGCATCAACC	
L211A-F	L211A	F	GGTTGATAGAGCTGCACGTGACGG	
L211A-R	L211A	R	CCGTCACGTGCAGCTCTATCAACC	
R212A-F	R212A	F	GGTTGATAGAGCTTTAGCTGACGG	
R212A-R	R212A	R	CCGTCAGCTAAAGCTCTATCAACC	
D213A-F	D213A	F	GATAGAGCTTTACGTGCAGGTTTC	
D213A-R	D213A	R	GAAACCTGCACGTAAAGCTCTATC	

G214A-F	G214A	F	GCTTTACGTGACGCTTTCAATCATTTAG	
G214A-R	G214A	R	CTAAATGATTGAAAGCGTCACGTAAAGC	
F215A-F	F215A	F	GCTTTACGTGACGGTGCAAATCATTTAG	
F215A-R	F215A	R	CTAAATGATTTGCACCGTCACGTAAAGC	
N216A-F	N216A	F	GCTTTACGTGACGGTTTCGCTCATTTAG	
N216A-R	N216A	R	CTAAATGAGCGAAACCGTCACGTAAAGC	
H217A-F	H217A	F	GACGGTTTCAATGCTTTAGATCGTGC	
H217A-R	H217A	R	GCACGATCTAAAGCATTGAAACCGTC	
L218A-F	L218A	F	GACGGTTTCAATCATGCAGATCGTGC	
L218A-R	L218A	R	GCACGATCTGCATGATTGAAACCGTC	
D219A-F	D219A	F	CATTTAGCTCGTGCTTTCAG	
D219A-R	D219A	R	CTGAAAGCACGAGCTAAATG	
R220A-F	R220A	F	CATTTAGATGCTGCTTTCAG	
R220A-R	R220A	R	CTGAAAGCAGCATCTAAATG	
F222A-F	F222A	F	GATCGTGCTGCAAGATACTATGG	
F222A-R	F222A	R	CCATAGTATCTTGCAGCACGATC	
R223A-F	R223A	F	CGTGCTTTCGCATACTATGG	
R223A-R	R223A	R	CCATAGTATGCGAAAGCACG	
Y224A-F	Y224A	F	GCTTTCAGAGCATATGGTGTAAAG	
Y224A-R	Y224A	R	CTTTACACCATATGCTCTGAAAGC	
Y225A-F	Y225A	F	GCTTTCAGATACGCTGGTGTAAAG	
Y225A-R	Y225A	R	CTTTACACCAGCGTATCTGAAAGC	
G226A-F	G226A	F	GCTTTCAGATACTATGCTGTAAAG	
G226A-R	G226A	R	CTTTACAGCATAGTATCTGAAAGC	
V227A-F	V227A	F	GATACTATGGTGCAAAGAAAGCCGC	
V227A-R	V227A	R	GCGGCTTTCTTTGCACCATAGTATC	
K228A-F	K228A	F	GGTGTAGCAAAAGCCGCTGATCTCG	
K228A-R	K228A	R	CGAGATCAGCGGCTTTTGCTACACC	
K229A-F	K229A	F	GGTGTAAAGGCAGCCGCTGATCTCG	
K229A-R	K229A	R	CGAGATCAGCGGCTGCCTTTACACC	
D232A-F	D232A	F	GGTGTAAAGAAAGCCGCTGCTCTCG	
D232A-R	D232A	R	CGAGAGCAGCGGCTTTCTTTACACC	
L233A-F	L233A	F	GCCGCTGATGCAGTTGTAATCTTGGAG	
L233A-R	L233A	R	CTCCAAGATTACAACTGCATCAGCGGC	

V234A-F	V234A	F	GCCGCTGATCTCGCTGTAATCTTGGAG	
V234A-R	V234A	R	CTCCAAGATTACAGCGAGATCAGCGGC	
V235A-F	V235A	F	GCCGCTGATCTCGTTGCAATCTTGGAG	
V235A-R	V235A	R	CTCCAAGATTGCAACGAGATCAGCGGC	
I236A-F	I236A	F	GCCGCTGATCTCGTTGTAGCATTGGAG	
I236A-R	I236A	R	CTCCAATGCTACAACGAGATCAGCGGC	
L237A-F	L237A	F	GCTGATCTCGTTGTAATCGCAGAGAAG	
L237A-R	L237A	R	CTTCTCTGCGATTACAACGAGATCAGC	
E238A-F	E238A	F	CTTGGCAAAGGCTTCTACTGCC	
E238A-R	E238A	R	GGCAGTAGAAGCCTTTGCCAAG	
K239A-F	K239A	F	CTTGGAGGCAGCTTCTACTGCC	
K239A-R	K239A	R	GGCAGTAGAAGCTGCCTCCAAG	
S241A-F	S241A	F	GAAGGCTGCTACTGCCTTGAAAGAAGC	
S241A-R	S241A	R	GCTTCTTTCAAGGCAGTAGCAGCCTTC	
T242A-F	T242A	F	GAAGGCTTCTGCTGCCTTGAAAGAAGC	
T242A-R	T242A	R	GCTTCTTTCAAGGCAGCAGAAGCCTTC	
L244A-F	L244A	F	GAAGGCTTCTACTGCCGCAAAAGAAGC	
L244A-R	L244A	R	GCTTCTTTTGCGGCAGTAGAAGCCTTC	
K245A-F	K245A	F	GCCTTGGCAGAAGCTGAACAAG	
K245A-R	K245A	R	CTTGTTCAGCTTCTGCCAAGGC	
E246A-F	E246A	F	GCCTTGAAAGCAGCTGAACAAG	
E246A-R	E246A	R	CTTGTTCAGCTGCTTTCAAGGC	
E248A-F	E248A	F	GCCTTGAAAGAAGCTGCACAAG	
E248A-R	E248A	R	CTTGTGCAGCTTCTTTCAAGGC	
Q249A-F	Q249A	F	GCTGAAGCAGAAACTGTAACC	
Q249A-R	Q249A	R	GGTTACAGTTTCTGCTTCAGC	
E250A-F	E250A	F	GCTGAACAAGCAACTGTAACC	
E250A-R	E250A	R	GGTTACAGTTGCTTGTTCAGC	
T251A-F	T251A	F	CAAGAAGCTGTAACCTTACTCACTCCTTTC	
T251A-R	T251A	R	GAAAGGAGTGAGTAAGGTTACAGCTTCTTG	
V252A-F	V252A	F	CAAGAAACTGCAACCTTACTCACTCCTTTC	
V252A-R	V252A	R	GAAAGGAGTGAGTAAGGTTGCAGTTTCTTG	
T253A-F	T253A	F	CAAGAAACTGTAGCATTACTCACTCCTTTC	
T253A-R	T253A	R	GAAAGGAGTGAGTAATGCTACAGTTTCTTG	

L254A-F	L254A	F	CAAGAAACTGTAACCGCACTCACTCCTTTC	
L254A-R	L254A	R	GAAAGGAGTGAGTGCGGTTACAGTTTCTTG	
L255A-F	L255A	F	CAAGAAACTGTAACCTTAGCAACTCCTTTC	
L255A-R	L255A	R	GAAAGGAGTTGCTAAGGTTACAGTTTCTTG	
T256A-F	T256A	F	CAAGAAACTGTAACCTTACTCGCTCCTTTC	
T256A-R	T256A	R	GAAAGGAGCGAGTAAGGTTACAGTTTCTTG	
P257A-F	P257A	F	CTCACTGCTTTCTTTAGACCAC	
P257A-R	P257A	R	GTGGTCTAAAGAAAGCAGTGAG	
F258A-F	F258A	F	CTCACTCCTGCATTTAGACCAC	
F258A-R	F258A	R	GTGGTCTAAATGCAGGAGTGAG	
F259A-F	F259A	F	CTCACTCCTTTCGCTAGACCAC	
F259A-R	F259A	R	GTGGTCTAGCGAAAGGAGTGAG	
R260A-F	R260A	F	CTCCTTTCTTTGCACCACAC	
R260A-R	R260A	R	GTGTGGTGCAAAGAAAGGAG	
P261A-F	P261A	F	CTTTAGAGCACACAATATTCAACTC	
P261A-R	P261A	R	GAGTTGAATATTGTGTGCTCTAAAG	
H262A-F	H262A	F	CTTTAGACCAGCAAATATTCAACTC	
H262A-R	H262A	R	GAGTTGAATATTTGCTGGTCTAAAG	
N263A-F	N263A	F	CTTTAGACCACACGCTATTCAACTC	
N263A-R	N263A	R	GAGTTGAATAGCGTGTGGTCTAAAG	
I264A-F	I264A	F	CTTTAGACCACACAATGCTCAACTC	
I264A-R	I264A	R	GAGTTGAGCATTGTGTGGTCTAAAG	
Q265A-F	Q265A	F	CACAATATTGCACTCATTCGTAATAC	
Q265A-R	Q265A	R	GTATTACGAATGAGTGCAATATTGTG	
L266A-F	L266A	F	CACAATATTCAAGCAATTCGTAATAC	
L266A-R	L266A	R	GTATTACGAATTGCTTGAATATTGTG	
I267A-F	I267A	F	CACAATATTCAACTCGCTCGTAATAC	
I267A-R	I267A	R	GTATTACGAGCGAGTTGAATATTGTG	
R268A-F	R268A	F	CTCATTGCTAATACTTTTGC	
R268A-R	R268A	R	GCAAAAGTATTAGCAATGAG	
N269A-F	N269A	F	CTCATTCGTGCTACTTTTGC	
N269A-R	N269A	R	GCAAAAGTAGCACGAATGAG	
T270A-F	T270A	F	CTCATTCGTAATGCTTTTGC	
T270A-R	T270A	R	GCAAAAGCATTACGAATGAG	

F271A-F	F271A	F	CGTAATACTGCTGCATTTTTGGG	
F271A-R	F271A	R	CCCAAAAATGCAGCAGTATTACG	
F273A-F	F273A	F	CGTAATACTTTTGCAGCTTTGGGTTC	
F273A-R	F273A	R	GAACCCAAAGCTGCAAAAGTATTACG	
L274A-F	L274A	F	CTTTTGCATTTGCAGGTTCTTTAGAC	
L274A-R	L274A	R	GTCTAAAGAACCTGCAAATGCAAAAG	
G275A-F	G275A	F	GCATTTTTGGCTTCTTTAGACTTTTTC	
G275A-R	G275A	R	GAAAAAGTCTAAAGAAGCCAAAAATGC	
S276A-F	S276A	F	GCATTTTTGGGTGCTTTAGACTTTTTC	
S276A-R	S276A	R	GAAAAAGTCTAAAGCACCCAAAAATGC	
L277A-F	L277A	F	GCATTTTTGGGTTCTGCAGACTTTTTCAC	
L277A-R	L277A	R	GTGAAAAAGTCTGCAGAACCCAAAAATGC	
D278A-F	D278A	F	GGTTCTTTAGCATTTTTCAC	
D278A-R	D278A	R	GTGAAAAATGCTAAAGAACC	
F279A-F	F279A	F	CTTTAGACGCTTTCACTAAAGTTTGG	
F279A-R	F279A	R	CCAAACTTTAGTGAAAGCGTCTAAAG	
F280A-F	F280A	F	CTTTAGACTTTGCAACTAAAGTTTGG	
F280A-R	F280A	R	CCAAACTTTAGTTGCAAAGTCTAAAG	
T281A-F	T281A	F	CTTTAGACTTTTTCGCTAAAGTTTGG	
T281A-R	T281A	R	CCAAACTTTAGCGAAAAAGTCTAAAG	
K282A-F	K282A	F	CACTGCAGTTTGGGATGATTTGG	
K282A-R	K282A	R	CCAAATCATCCCAAACTGCAGTG	
V283A-F	V283A	F	CTTTTTCACTAAAGCTTGGGATGATTTG	
V283A-R	V283A	R	CAAATCATCCCAAGCTTTAGTGAAAAAG	
W284A-F	W284A	F	CACTAAAGTTGCAGATGATTTGG	
W284A-R	W284A	R	CCAAATCATCTGCAACTTTAGTG	
D285A-F	D285A	F	CACTAAAGTTTGGGCTGATTTGG	
D285A-R	D285A	R	CCAAATCAGCCCAAACTTTAGTG	
D286A-F	D286A	F	CACTAAAGTTTGGGATGCTTTGG	
D286A-R	D286A	R	CCAAAGCATCCCAAACTTTAGTG	
L287A-F	L287A	F	GTTTGGGATGATGCAGAAATTGAAG	
L287A-R	L287A	R	CTTCAATTTCTGCATCATCCCAAAC	
E288A-F	E288A	F	CACTAAAGTTTGGGATGATTTGGCAATTG	
I289A-F	I289A	F	CACTAAAGTTTGGGATGATTTGGAAGCTGA AGATGATTTATTC	

E290A-F	E290A	F	CACTAAAGTTTGGGATGATTTGGAAATTGCA GATG	
D291A-F	D291A	F	CACTAAAGTTTGGGATGATTTGGAAATTGAA GCTGATTTATTC	
D292A-F	D292A	F	CACTAAAGTTTGGGATGATTTGGAAATTGAA GATGCTTTATTC	
L293A-F	L293A	F	CACTAAAGTTTGGGATGATTTGGAAATTGAA GATGATGCATTCTTATTAATTAGTGC	
F294A-F	F294A	F	CACTAAAGTTTGGGATGATTTGGAAATTGAA GATGATTTAGCTTTATTAATTAGTGC	
L295A-F	L295A	F	CACTAAAGTTTGGGATGATTTGGAAATTGAA GATGATTTATTCGCATTAATTAGTGC	
L296A-F	L296A	F	CACTAAAGTTTGGGATGATTTGGAAATTGAA GATGATTTATTCTTAGCAATTAGTGC	
I297A-R	I297A	R	CACCACCTCCCTCGAGATAAATCAATTCAAT TTGAGTATATTTGTTAAGAGCACTAGCTAAT AAG	
S298A-R	S298A	R	CACCACCTCCCTCGAGATAAATCAATTCAAT TTGAGTATATTTGTTAAGAGCAGCAATTAAT AAG	
L300A-R	L300A	R	CACCACCTCCCTCGAGATAAATCAATTCAAT TTGAGTATATTTGTTAGCAGCACTAATTAAT AAG	
N301A-R	N301A	R	CACCACCTCCCTCGAGATAAATCAATTCAAT TTGAGTATATTTAGCAAGAGCACTAATTAAT AAG	
K302A-R	K302A	R	CACCACCTCCCTCGAGATAAATCAATTCAAT TTGAGTATATGCGTTAAGAGCAC	
Y303A-R	Y303A	R	CACCACCTCCCTCGAGATAAATCAATTCAAT TTGAGTAGCTTTGTTAAGAGCAC	
T304A-R	T304A	R	CACCACCTCCCTCGAGATAAATCAATTCAAT TTGAGCATATTTGTTAAGAGCAC	
Q305A-R	Q305A	R	CACCACCTCCCTCGAGATAAATCAATTCAAT TGCAGTATATTTGTTAAGAGCAC	
I306A-R	I306A	R	CACCACCTCCCTCGAGATAAATCAATTCAGC TTGAGTATATTTGTTAAGAGCAC	
E307A-R	E307A	R	CACCACCTCCCTCGAGATAAATCAATGCAAT TTGAG	
L308A-R	L308A	R	CACCACCTCCCTCGAGATAAATAGCTTCAAT TTGAGTATATTTG	
I309A-R	I309A	R	CACCACCTCCCTCGAGATAAGCCAATTCAAT TTGAGTATATTTG	
Y310A-R	Y310A	R	CACCACCTCCCTCGAGAGCAATCAATTCAAT TTGAGTATATTTG	
V163W-R	V163W	R	CTTTTTCCATGATCCAATCAATTCACGACCC TC	
V163W-F	V163W	F	GGATCATGGAAAAAGATCATTAAGAGAGTC TCAA	
I183W-R	I183W	R	CTTTAGCCAATTCTTCTCCATTTCATTTGCTT TC	
I183W-F	I183W	F	AAGAATTGGCTAAAGATTTTAATCAAAGTGT TTTTC	
L187W-R	L187W	R	TTGATCCAAATCTTTAGGATATTCTTCTCCAT TTC	
L187W-F	L187W	F	AAAGATTTGGATCAAAGTGTTTTTCTACATT GATTC	
L211W-R	L211W	R	GTCACGCCAAGCTCTATCAACCTTTGC	
L211W-F	L211W	F	AGAGCTTGGCGTGACGGTTTCAAT	
L296W-R	L296W	R	ACTAATCCATAAGAATAAATCATCTTCAATT TCCAAATCAT	
L296W-F	L296W	F	TTCTTATGGATTAGTGCTCTTAACAAATATA CTC	

L300W-R	L300W	R	CACCACCTCCCTCGAGATAAATCAATTCAAT TTGAGTATATTTGTTCCAAGCACT	
I309W-R	I309W	R	CACCACCTCCCTCGAGATACCACAATTCAAT TTGAGTATATTTGTT	
I152W-F	I152W	F	GGTTTAAAGAAATTGTGGCCAGAAGAG	
I152W-R	I152W	R	CTCTTCTGGCCACAATTTCTTTAAACC	
L159W-F	L159W	F	CGTGAATGGATTGGATCAGTTAAAAAG	
L159W-R	L159W	R	CTTTTTAACTGATCCAATCCATTCACG	
I160W-F	I160W	F	CGTGAATTGTGGGGATCAGTTAAAAAG	
I160W-R	I160W	R	CTTTTTAACTGATCCCCACAATTCACG	
I166W-F	I166W	F	CAGTTAAAAAGTGGATTAAGAGAGTC	
I166W-R	I166W	R	GACTCTCTTAATCCACTTTTTAACTG	
I186W-F	I186W	F	GGAGAAGAATATCCTAAAGTGGTTAATC	
I186W-R	I186W	R	GATTAACCACTTTAGGATATTCTTCTCC	
V190W-F	V190W	F	GATTTTAATCAAATGGTTTTTCTAC	
V190W-R	V190W	R	GTAGAAAAACCATTTGATTAAAATC	
L204W-F	L204W	F	GCAATTCAAATTGGTGATTGGGCAAAGG	
L204W-R	L204W	R	CCTTTGCCCAATCACCAATTTGAATTGC	
V207W-F	V207W	F	GATTTGGCAAAGTGGGATAGAGC	
V207W-R	V207W	R	GCTCTATCCCACTTTGCCAAATC	
L218W-F	L218W	F	GACGGTTTCAATCATTGGGATCGTGC	
L218W-R	L218W	R	GCACGATCCCAATGATTGAAACCGTC	
L244W-F	L244W	F	GAAGGCTTCTACTGCCTGGAAAGAAGC	
L244W-R	L244W	R	GCTTCTTTCCAGGCAGTAGAAGCCTTC	
L254W-F	L254W	F	CAAGAAACTGTAACCTGGCTCACTCCTTTC	
L254W-R	L254W	R	GAAAGGAGTGAGCCAGGTTACAGTTTCTTG	
L274W-F	L274W	F	CTTTTGCATTTTGGGGTTCTTTAGAC	
L274W-R	L274W	R	GTCTAAAGAACCCCAAAATGCAAAAG	
V283W-F	V283W	F	CTTTTTCACTAAATGGTGGGATGATTTG	
V283W-R	V283W	R	CAAATCATCCCACCATTTAGTGAAAAAG	
L293W-F	L293W	F	CACTAAAGTTTGGGATGATTTGGAAATTGAA GATGATTGGTTCTTATTAATTAGTGC	
I297W-R	I297W	R	CACCACCTCCCTCGAGATAAATCAATTCAAT TTGAGTATATTTGTTAAGAGCACTCCATAAT AAG	
I306W-R	I306W	R	CACCACCTCCCTCGAGATAAATCAATTCCCA TTGAGTATATTTGTTAAGAGCAC	
L308W-R	L308W	R	CACCACCTCCCTCGAGATAAATCCATTCAAT TTGAGTATATTTG	
L293W-R	L293W	R	TCCAAATCATCCCAAACTTTAGTGAAAAAGT CTAAAG	

Supplementary References

1. Larkin, M. A. *et al.* Clustal W and Clustal X version 2.0. *Bioinformatics* **23**, 2947-2948 (2007).
2. Keep, N. H. *et al.* A modulator of rho family G proteins, rhoGDI, binds these G proteins via an immunoglobulin-like domain and a flexible N-terminal arm. *Structure* **5**, 623-633 (1997).
3. Hoffman, G. R., Nassar, N. & Cerione, R. A. Structure of the Rho family GTP-binding protein Cdc42 in complex with the multifunctional regulator RhoGDI. *Cell* **100**, 345-356 (2000).
4. Grizot, S. *et al.* Crystal structure of the Rac1-RhoGDI complex involved in NADPH oxidase activation. *Biochemistry* **40**, 10007-10013 (2001).
5. Tnimov, Z. *et al.* Quantitive anlysis of prenylated RhoA interaction with its chaperone, RhoGDI. *J. Biol. Chem.* **287**, 26549-26562 (2012).
6. Scheffzek, K., Stephan, I., Jensen, O. N., Illenberger, D. & Gierschik, P. The Rac-RhoGDI complex and the structural basis for the regulation of Rho proteins by RhoGDI. *Nat. Struct. Biol.* **7**, 122-126 (2000).
7. Hanzal-Bayer, M., Renault, L., Roversi, P., Wittinghofer, A. & Hillig, R. C. The complex of Arl2-GTP and PDEδ: from structure to function. *EMBO J.* **21**, 2095-2106 (2002).
8. Ismail, S. A. *et al.* Arl2-GTP and Arl3-GTP regulate a GDI-like transport system for farnesylated cargo. *Nat. Chem. Biol.* **7**, 942-949 (2011).
9. Wätzlich, D. *et al.* The interplay between RPGR, PDEd and Arl2/3 regulate the ciliary targeting of farnesylated cargo. *EMBO Rep.* **14**, 465-472 (2013).
10. Dharmaiah, S. *et al.* Structural basis of recognition of farnesylated and methylated KRAS4b by PDEδ. *Proc. Natl. Acad. Sci. U.S.A.* **113**, E6766-E6775 (2016).
11. Fansa, E. K., Kösling, S. K., Zent, E., Wittinghofer, A. & Ismail S. PDE6d-mediated sorting of INPP5E into the cilium is determined by

cargo-carrier affinity. *Nat. Commun.* **7**, 11366 (2016).

12. Qureshi, B. M. *et al.* Mechanistic insights into the role of prenyl-binding protein PrBP/δ in membrane dissociation of phosphodiesterase 6. *Nat. Commun.* **9**, 90 (2018).
13. Zhang, H. *et al.* UNC119 is required for G protein trafficking in sensory neurons. *Nat. Neurosci.* **14**, 874-880 (2011).
14. Ismail, S. A. *et al.* Structural basis for Arl3-specific release of myristoylated ciliary cargo from UNC119. *EMBO J.* **31**, 4085-4094 (2012).
15. Jaiswal, M. *et al.* Novel biochemical and structural insights into the interaction of myristoylated cargo with Unc119 protein and their release by Arl2/3. *J. Biol. Chem.* **291**, 20766-20778 (2016).
16. Schalk, I. *et al.* Structure and mutational analysis of Rab GDP-dissociation inhibitor. *Nature* **381**, 42-48 (1996).
17. An, Y. *et al.* Geranylgeranyl switching regulates GDI-Rab GTPase recycling. *Structure* **11**, 347-357 (2003).
18. Rak, A. *et al.* Structure of Rab GDP-dissociation inhibitor in complex with prenylated YPT1 GTPase. *Science* **302**, 646-650 (2003).
19. Pylypenko, O. *et al.* Structure of doubly prenylated Ypt1:GDI complex and the mechanism of GDI-mediated Rab recycling. *EMBO J.* **25**, 13-23 (2006).
20. Zhang, X. *et al.* Crystal structure of TIPE2 provides insights into immune homeostasis. *Nat. Struct. Mol. Biol.* **16**, 89-90 (2009).
21. Fayngerts, S. A. *et al.* TIPE3 is the transfer protein of lipid second messengers that promote cancer. *Cancer Cell* **26**, 465-478 (2014).
22. Kim, J.-S. *et al.* The Tnfaip8-PE complex is a novel upstream effector in the anti-autophagic action of insulin. *Sci. Rep.* **7**, 6248 (2017).
