# Supplementary material for: Structural basis of Gip1 for cytosolic sequestration of G protein in wide-range chemotaxis
Source: Nat Commun. 2018 Nov 6;9:4635. doi: 10.1038/s41467-018-07035-x (PMC6219514; doi:10.1038/s41467-018-07035-x)
Supplement: Supplementary file 9 — Description of Additional Supplementary Files [file 41467_2018_7035_MOESM9_ESM.docx]

**Title:** Supplementary Movie 1.
**Description:** Overall structure of Gip1(146-310) accommodating the phospholipid. The structures of Gip1(146-310) and phospholipid (PDB 5Z1N) are represented by a surface model and a CPK model, respectively. The surface of the cavity is shown in grey.

**Title:** Supplementary Movie 2.
**Description:** Residues surrounding the phospholipid. The structure of Gip1(146-310) is represented by a cartoon model with side chains shown as stick models within van der Waal’s distance from the phospholipid. The phospholipid is shown as a stick model with the 2mFo-DFc electron density map contoured at 1.0 σ.

**Title:** Supplementary Movie 3.
**Description:** Chemotaxis of gip1Δ (vector) cells. Cells were placed in a 100 µM cAMP gradient applied with a central pipette. Images are shown at 1-min intervals for 120 min. Scale bar, 50 µm.

**Title:** Supplementary Movie 4.
**Description:** Chemotaxis of gip1Δ wild-type Gip1-GFPF (WT) cells. Cells were placed in a 100 µM cAMP gradient applied with a central pipette. Images are shown at 1-min intervals for 120 min. Scale bar, 50 µm.

**Title:** Supplementary Movie 5.
**Description:** Chemotaxis of gip1Δ mutant Gip1-GFPF (D208A) cells. Cells were placed in a 100 µM cAMP gradient applied with a central pipette. Images are shown at 1-min intervals for 120 min. Scale bar, 50 µm.

**Title:** Supplementary Movie 6.
**Description:** Chemotaxis of gip1Δ mutant Gip1-GFPF (ΔC-tail) cells. Cells were placed in a 100 µM cAMP gradient applied with a central pipette. Images are shown at 1-min intervals for 120 min. Scale bar, 50 µm.
